# CRAM 3.1: advances in the CRAM file format

**DOI:** 10.1093/bioinformatics/btac010

**Published:** 2022-01-06

**Authors:** James K Bonfield

**Affiliations:** Informatics and Digital Solutions, Wellcome Sanger Institute, Wellcome Genome Campus, Hinxton CB10 1SA, UK

## Abstract

**Motivation:**

CRAM has established itself as a high compression alternative to the BAM file format for DNA sequencing data. We describe updates to further improve this on modern sequencing instruments.

**Results:**

With Illumina data CRAM 3.1 is 7–15% smaller than the equivalent CRAM 3.0 file, and 50–70% smaller than the corresponding BAM file. Long-read technology shows more modest compression due to the presence of high-entropy signals.

**Availability and implementation:**

The CRAM 3.0 specification is freely available from https://samtools.github.io/hts-specs/CRAMv3.pdf. The CRAM 3.1 improvements are available in a separate OpenSource HTScodecs library from https://github.com/samtools/htscodecs, and have been incorporated into HTSlib.

**Supplementary information:**

Supplementary data are available at *Bioinformatics* online.

## 1 Introduction

It has been well established that the growth in genomic sequencing data is challenging (Stephens *et al.*, 2015). The earlier file formats of SAM and BAM (Li *et al.*, 2009) were appropriate for the era, but better techniques were soon required. The notion of reference-based compression, storing only the differences between DNA sequence fragments and the reference they have been aligned against, was proposed (Fritz *et al.*, 2011). Fritz *et al.* also proposed techniques for efficient encoding of unaligned data by the use of sequence assembly to generate consensus sequences, which may then be used as the reference sequence to compare against. This work leads to the development of CRAM by the European Bioinformatics Institute (Cochrane *et al.*, 2013).

The primary goals of CRAM were a reduction in storage requirements, while maintaining direct compatibility with BAM, permitting lossless round trips. All data representable in BAM are also available in CRAM. This includes the SAM header, which is the same format in CRAM, and the optional auxiliary key-value ‘tags’. These annotations are defined by a shared SAMtags specification (https://samtools.github.io/hts-specs/SAMtags.pdf).

Although reference compression is where the original work focussed, it is wrong to assume that this is the primary reason for CRAM’s reduced file size. BAM serializes all data together (first name, chromosome, position, sequence, quality and auxiliary fields, then second name, chromosome and so on). This leads to poor compression ratios as names, sequences and quality values all have very different characteristics. CRAM has a column-oriented approach, where a block of names are compressed together or a block of qualities together. Each block can be compressed with an algorithm specific to that data type. This leads to significantly reduced file sizes and is often the biggest factor in file reduction.

The first tool implementing CRAM (then version 1.0) was CRAMtools (Vadim Zalunin, 2011, unpublished data), written in Java. The Scramble tool (Bonfield, 2014) was the first C implementation and lead to a specification tidy-up producing CRAM 2.0 in 2013. HTSlib (Bonfield *et al.*, 2021) gained CRAM support shortly after. CRAM 3.0 appeared a year later in 2014, with some additional compression codecs including the rANS entropy encoder (Duda, 2013) and LZMA (Lempel Ziv Markov-chain Algorithm, Igor Pavlov, 1998, unpublished data). More implementations of CRAM have since appeared, written in JavaScript (Buels *et al.*, 2019) and Rust (https://github.com/zaeleus/noodles). Many more programming languages support CRAM via bindings to one of these existing implementations.

The CRAM specification is now maintained by the Global Alliance for Genomics and Health (GA4GH: https://www.ga4gh.org/cram/). It ties in with a number of other GA4GH standards and protocols (Rehm *et al.*, 2021), which further extend the features and capabilities. Reference sequences may be obtained either via local files or using a refget server (Yates *et al.*, 2021). CRAM files can be streamed remotely using the htsget protocol (Kelleher *et al.*, 2019), and they may be encrypted using Crypt4GH (Senf *et al.*, 2021).

Since 2014 CRAM has been very stable, but a lot has changed data-wise. Illumina’s quality values have been successively quantized from 40 discrete values, to 8, and now with NovaSeq to 4 (Illumina, 2012). We have also seen the rise of long-read technologies and more complex auxiliary data types being embedded in the files. As the data changes, so too should the encoding and compression methods available to the format. Methods, such as Run-Length Encoding (RLE), were considered and explicitly rejected as unhelpful in the original CRAM development, but now these same techniques can be beneficial. CRAM 3.1 is the first major update to CRAM since 2014. It keeps the underlying format unchanged, but adds new compression codecs. This is the first CRAM version to include data-type-specific codecs (for identifiers and quality scores), rather than relying on general purpose methods.

With large data volumes come large processing requirements. By default, CRAM optimizes for a balance between CPU cost, file size and granularity of random access. However, the option of higher memory and CPU requirements for long-term archival is still worthy of consideration so CRAM 3.1 also improves support for archival modes.

At the time of writing CRAM 3.1 is in draft. Implementations of the new codecs exist in C (HTSlib, SAMtools and Scramble) with a JavaScript proof of concept.

## 2 Materials and methods

**Fig. 1.**
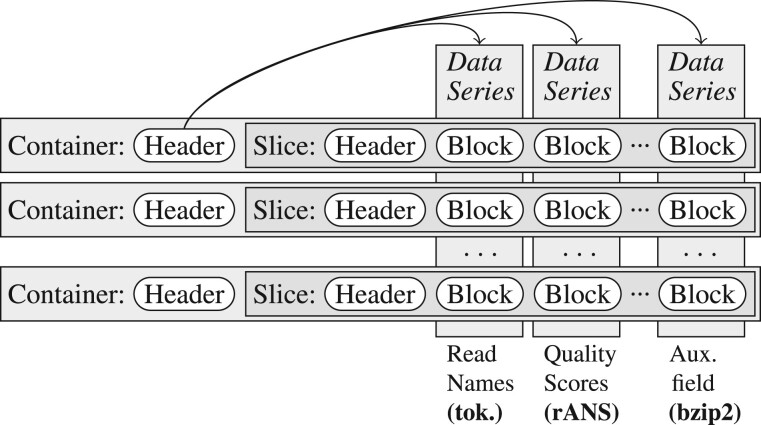
Logical layout of a CRAM file, showing containers and slices as rows, and data series as columns. Random access is possible on rows, with rapid filtering (discarding) of columns

The basic structure of CRAM can be seen in Figure 1. It starts with a header matching the SAM specification, although it mandates the use of MD5sums on reference sequence lines for data provenance and to ensure correct decoding.

CRAM’s records are broken down into data series, loosely fitting the columns in a SAM file, such as alignment position, quality values or CIGAR string components. Each auxiliary tag also gets its own data series.

The CRAM header is then followed by a series of containers, which in turn hold slices and data blocks within them. The container header consists of meta-data describing how and where each data series is encoded. The slices are collections of alignment records, applying the encoding rules described by the container to the records and storing the result in the requested blocks. The blocks are then compressed using their own selected compression algorithms. It is these algorithms, which have been added to in CRAM 3.1. Slices may be of any size, but the HTSlib implementation defaults to 10 000 records, or fewer if long reads are present.

The self-describing nature of CRAM offers great flexibility to the encoder, meaning that over time encoder improvements may yield smaller files. For example, the first 10 million reads of a NovaSeq alignment in CRAM 3.0 format produced by HTSlib 1.2 takes up 199.6 MB. The latest HTSlib 1.13 encodes the same file in 195.2 MB and Picard 2.25.7 with default options uses 254.1 MB. All of these files are compatible and have the same choice of codecs available. The algorithms for selecting which codec to use are implementation specific and outside of the scope of both the CRAM specification and this manuscript, however details for how HTSlib adapts and learns the codecs to use are available in the Supplementary Material.

As it is possible to store each data series in its own block, this permits selective decoding where only specific types of data need to be decoded. This can be of great benefit to certain algorithms. For example, the ‘samtools flagstat’ command gives a summary of SAM FLAG bit frequencies (Danecek *et al.*, 2021). Although it does not need to know about sequences, quality values or read identifiers, with a BAM file it is still required to decompress this data due to the serial nature of the format. With CRAM it only decompresses the data series required. Consequentially ‘samtools flagstat’ on the NA12878 Platinum Genomes file takes 7 min 5 s on the CRAM file and 22 min 52 s on the BAM file (with neither file in disc cache).

The NM and MD SAM auxiliary tags have special handling within CRAM. As they describe the difference between an aligned sequence and the reference and we are typically doing reference-based compression, they may be omitted and generated on-the-fly during decode. If these values are found to be in error then they can either be corrected, or if we wish to have bug-compatible data then the (incorrect) values may be stored verbatim in the CRAM file.

Each slice can optionally also contain a copy of the reference used for that genomic region. This permits CRAM to do reference-based compression while removing the dependency on external data files. For deeply covered regions this does not have a significant impact on compression ratios. This embedded reference could be a consensus rather than the official external reference, offering the potential for improved compression via fewer sequence differences. However, doing so means storing NM and MD verbatim in regions where consensus and reference differ, negating most of the gains. Note, this problem is resolved in Deez (Hach *et al.*, 2014) by using a two-level delta (sequence to consensus and consensus to reference), and may be considered for a future CRAM update.

CRAM distinguishes between data encodings (what data series goes where and in what byte layout) as described by the container header, and compression codecs (what algorithm is used to compress the data blocks), which is described in the blocks themselves. The compression codecs permitted in CRAM 3.0 are three external general compression tools—deflate (Deutsch and Gailly, 1996), bzip2 and LZMA—and the rANS entropy encoder. The entropy encoder uses static frequencies, written at the start of the block, which can be either Order-0 or Order-1 metrics. An example of Order-0 frequencies is the observation that the letter ‘u’ accounts for 3% of the total letter usage in English, while an Order-1 observation is that ‘u’ occurs nearly 100% of the time following the letter ‘q’.

The current official CRAM format is 3.0, but CRAM 3.1 has been a draft standard since 2019. The layout of the CRAM format is unchanged, but new custom compression codecs have been added. These include:

**Improved rANS with data transformations:** The reduction in the count of discrete quality values in Illumina data from 40 (early HiSeq) to 4 (NovaSeq) has meant that some simple data transformations can reduce both data size and time to encode. The updated rANS codec now performs 16-bit state renormalization instead of the earlier 8-bit step, for faster processing. Additionally, the four interleaved rANS states can now optionally be expanded to 32 states. Typically this is only applied on large blocks due to the extra overhead of storing an additional 28 states. This permits multiple efficient SIMD implementations (plus a non-SIMD ‘scalar’ version), all binary compatible with each other. Our software automatically selects the implementation based on the processor available.

Additionally, data transformations can now be applied before entropy encoding:

**RLE:** RLE uses a Mespotine RLE variant (Mespotine, 2015), which stores a list of symbols for which runs are always stored (even if the additional run-length is zero). Symbols not in this list will never have a run-length attached (even if long). This is highly effective at reducing the size of data with stationary probabilities, such as quality values.

The symbol literals and run-lengths are separated into two distinct data streams, each of which is compressed independently using rANS before concatenation together.

**PACK:** The bit-packing strategy initially counts the number of distinct symbols in the input data. If it is 2 or less, then each value is assigned 1 bit and we pack 8 into a byte, if it is 4 or less, each value consumes 2 bits (4 to a byte) and if it is 16 or less then each value consumes 4 bits (2 to a byte). The data stream starts with a small lookup table mapping symbols to bit-values. This is an extremely rapid way to reduce the data volume, speeding up the relatively slower entropy encoding part. It many cases it also boosts the compression ratio.

**STRIPE:** This de-interleaves data into N separate streams consisting of bytes at location *Nx* + *c* for 0 ≤ *c* < *N*. For example, with *N* = 4 bytes 0,4,8,…, 1,5,9,…, 2,6,10,… and 3,7,11,… produce four streams. Each stream is then compressed using rANS, potentially also including further RLE and PACK transforms.

Both RLE and PACK are highly effective at compressing Illumina’s 4-quantized quality values from the NovaSeq platform, while STRIPE is useful for 16-bit and 32-bit integer values. The name tokenizer (below) also makes extensive use of the rANS data transformations.

**Adaptive arithmetic encoder:** This is a byte-wise arithmetic codec with adaptively updated frequencies. This helps for data types with non-stationary probability distributions, but it has a significantly higher CPU overhead than rANS. It also includes the data transformations used in the updated rANS codec. However, the RLE method is more complex, using per symbol models of run-lengths instead of separating into literal and run-length streams.

The adaptive arithmetic encoder is internally used by the FQZComp quality codec and optionally by the name tokenizer. It has Order-0 and Order-1 models, but being adaptive in nature (without the need to store large frequency tables) it could trivially be extended to support higher order models if deemed necessary in the future. These have not been added to date due to the usual desire to encode small blocks to permit fine-grained random access.

**FQZComp quality encoder:** This is a generalized version of the quality model used in the FQZComp tool (Bonfield and Mahoney, 2013). It is currently limited to a maximum of 16 bits of context in order to permit rapid model tuning and to make it appropriate for a more random-access-oriented file format. The context is applied to an adaptive arithmetic encoder. This makes it slower than rANS and more suitable to data archival. The construction of this context is flexible, offering great opportunity for learning and tuning to a specific dataset. The data format includes the model description, so changing models will not produce incompatible CRAM files. A variety of pre-defined models are used by our implementation, but this is something we expect to improve upon.

Data available for model context generation include:

**Previous quality values:** shifted and bitwise-ORed together. Qualities can also be indirected via a lookup table, e.g. to first reduce the NovaSeq qualities to 2 bits.

**Base** **position:** the location along the current read (left to right). When coupled with a position lookup table this permits selection of specific cycles, exploiting any knowledge of good and bad cycles.

**Cumulative difference:** a counter that increments every time a quality value differs to the previous one, optionally quantized via a lookup table. The variation in quality values along a read is often indicative of future variation.

**Selector bits:** unlike the quality derived metrics above, the selector bits are copied verbatim from the data stream into the context. These are used for any data the encoder wishes to use to separate qualities values into discrete groups. Examples could be splitting by average quality value or clustering by *x*/*y* location extracted from the read name (e.g. flowcell edge effects or bubbles). This provides a lot of flexibility for future quality classification and clustering methods.

There is scope for extending this to also include the adjacent sequence bases, for further improvement in PacBio and ONT quality encoding, but this would add interdependence between CRAM data series so such a change is delayed to CRAM 4.0.

Additional reverse complement and duplicate record flags are used to control the orientation of the quality string or to copy a previous quality string. The description of how to compute the model context is written into the quality stream, meaning the decoder follows instructions written by the encoder, permitting considerable flexibility in encoder improvements.

**Fig. 2.**
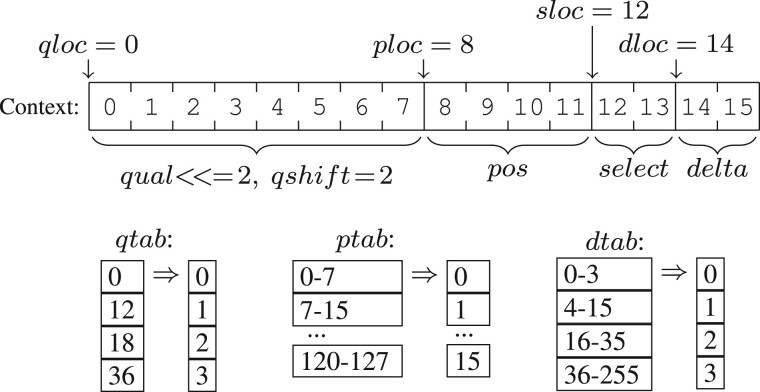
An example FQZComp configuration describing how previous quality values, the position in the sequence, a running sum of the quality differences (delta) and a generic model selector can be combined with lookup tables to generate a context model

An example FQZComp configuration is shown in Figure 2.

**Read name tokenizer:** The read identifiers are often highly structured, such as ‘HSQ1004:134:C0D8DACXX:4:2107:20375:180666’. Much like how CRAM separates the primary SAM fields into columns, the name tokenizer separates the components of a read name. A tokenized prior identifier is used to compare against and the tokens are encoded in the context of these previous token values. Token types are:

**CHAR:** A single character.

**ALPHA:** A string of multiple characters.

**DIGITS:** A series of digits, up to 10 long. Longer runs of digits are broken down into multiple DIGITS tokens.

**DIGITS0:** A series of digits starting with 0 or more leading zeros.

**MATCH:** A flag to indicate this token exactly matches the same token position in the prior identifier.

**DDELTA:** Numeric delta against the number in the same tokenized column in the prior identifier. The delta must be between 0 and 255.

**DDELTA0:** Numeric delta with leading zeros. The number of leading zeros must match. The delta must be between 0 and 255. For example, 0123 and 0130 would use DDELTA0 7.

**NOP:** A token, which does nothing. This is used to ensure the number of tokens is consistent between identifiers, or to separate sets of token columns apart when two very different styles of read identifiers are mixed in the same input file.

**END:** A marker for the last token.

These token types and their associated values are each stored in their own column-specific data streams, potentially producing a dozen or more streams. Each stream is then compressed using rANS or adaptive arithmetic codecs with their results then serialized together.

The origins of this method also come from the FQZComp tool, although similar strategies were employed by many of the other SequenceSqueeze entries and subsequent derivations, with broadly similar results. This tokenizer was also successfully submitted to MPEG-G, hence the same tokenization scheme is used there (coupled to a different entropy encoder).

CRAM also permits some controlled data loss. Read names may be discarded, with new names generated during decode. Read pairing within a slice is encoded by explicit links between records, so the generated names are still in pairs. Quality values may also be omitted, with only specific sites being stored. However, this is not the recommended approach to quality value reduction. It is possible to reduce the entropy of quality value either by smoothing methods, such as P-block (Cánovas *et al.*, 2014) or site-specific quality reassignment via CALQ (Voges *et al.*, 2018) or Crumble (Bonfield *et al.*, 2019). These modified quality strings are then much more compressible, particularly, when combined with the newer RLE rANS transform. As such, we view quality loss best dealt with as a topic external to the file format.

The primary focus of CRAM is with sorted aligned data. However, SAM, BAM and CRAM all support unaligned data too and the addition of both per-file and per-read meta-data arguably make these a superior format to using FASTQ. There are many FASTQ compression tools, which offer superior ratios to unaligned CRAM, but our approach to FASTQ is primarily as a transitional format between sequencing and either alignment or assembly rather than as a suitable long-term archival format. That said, combining an approximate rapid sequence aligner with CRAM can be used to reduce data size. Examples of this using SNAP (Zaharia *et al.*, 2011) are in the Supplementary Material.

CRAM may also store non-position-sorted data, such as sorted by read name order. Sequences may use either reference-based or reference-less encoding, with the latter sometimes being a more time and memory performant option when dealing with unsorted data from very large genomes.

## 3 Results

This manuscript is on improvements to the CRAM format rather than a specific implementation, however, it is not possible to analyse the performance of the format without evaluating an implementation. We use SAMtools and HTSlib 1.13. Where possible, we also compare against Deez, MPEG-G and Genozip (Lan *et al.*, 2021). The GenomSys MPEG-G tool is not freely available so figures reported are taken from their paper and its references. Note, their CRAM figures significantly differ to ours. We requested clarification from the authors, but received no reply and some of these differences remain unexplained.

Benchmarks for the new codecs in isolation, outside of CRAM, can be seen in the tables below.

Table 1 shows entropy encoder speeds for the first 1 million records from NovaSeq data. Speeds on HiSeq 2000 qualities are listed in the Supplementary Material. Also shown are other static frequency entropy encoders including Zlib’s Huffman encoder and the Finite State Entropy (FSE) implementation used in Zstd (Collet, 2021). These only support Order-0 encoding and Huffman is unable to encode the skewed 4-quality NovaSeq data efficiently.

**Table 1.** NovaSeq quality: entropy encoder speeds

Program	Option	Size (MB)	Enc (MB/s)	Dec (MB/s)
Zlib	Huffman	21.48	151	365
FSE	tANS	9.90	435	531
FSE	Huffman	20.74	710	1303
rANS4x8	O0	9.91	405	624
rANS4x16	O0	9.90	426	797
rANS32x16-AVX2	O0	9.92	709	1949
rANS4x8	O1	9.14	291	408
rANS4x16	O1	9.14	304	608
rANS32x16-AVX2	O1	9.16	430	1484
rANS32x16-AVX512	O0, PACK+RLE	9.17	627	1302
rANS32x16-AVX512	O1, PACK+RLE	8.26	533	1078
arith	O0	9.83	120	94
arith	O0, PACK+RLE	9.16	220	188
arith	O1	9.12	105	91
arith	O1, PACK+RLE	8.12	156	115
GABAC-app	−d15 -s1	8.40	5	8

*Note*: Block size 1 MB, except FSE Huffman, which used 128 KB. Figures produced using the unroll32 branch (git hash 9e9c3f7) from https://github.com/jkbonfield/htscodecs. The test system self-reported as "Intel(R) Xeon(R) Gold 6242 CPU @ 2.80GHz". (Note this differs to the full CRAM benchmarks, due to the desite to include AVX2 and AVX512 figures.)

This table also shows performance of the adaptive coder along with the Genomic Adaptive Binary Arithmetic Coder (GABAC) (Voges *et al.*, 2020) used in the OpenSource Genie (Bliss *et al.*, 2018) reference implementation of MPEG-G (Voges *et al.*, 2021). This latter provides a reasonable ratio for the low-entropy NovaSeq dataset, but is two orders of magnitude slower than the faster methods. Note, this may not be indicative of a well optimized GABAC implementation.

Table 2 shows the performance of two pre-defined FQZComp configurations on high-entropy HiSeq 2000 quality values and low-entropy NovaSeq quality values, compared against libbsc and the original FQZComp FASTQ compression tool. It can be seen that the choice of model configuration can be critical, as well as demonstrating advances in the FQZComp parameters made since the original release. This particular set of HiSeq 2000 data has some highly erroneous cycles, so using more bits to track position within the read is very productive. Additionally, this model utilizes the embedded selector bits to separate data by READ1 and READ2 flags, slightly improving the compression of the NovaSeq data too.

**Table 2.** Quality value FQZComp performance

Program	Option	Size (MB)	Enc (MB/s)	Dec (MB/s)
NovaSeq qualities				
bsc-3.1.7	−m0e2tTp	7.72	19.3	35.8
FQZComp-4.6	−q2	8.12	46.2	40.8
FQZComp-4.6	−q3	7.72	41.9	37.8
CRAM FQZComp	−s0	7.27	28.6	51.8
CRAM FQZComp	−s1+read 1/2	7.21	21.4	26.7
HiSeq 2000 qualities				
bsc-3.1.7	−m0e2tTp	42.6	6.5	9.4
FQZComp-4.6	−q2	44.9	23.3	20.0
FQZComp-4.6	−q3	41.0	19.6	16.7
CRAM FQZComp	−s0	42.5	15.8	17.8
CRAM FQZComp	−s1+read 1/2	31.3	13.3	14.9

*Note*: Compression of NovaSeq and HiSeq 2000 quality values using libbsc, CRAM 3.1’s FQZComp and the original FQZComp-4.6 tool, in 10 blocks of 100 000 records. This uses the same htscodecs branch and test system as the rANS benchmark in Table 1.

Table 3 shows the performance of the name tokenizer on 10 blocks each containing 100 000 NovaSeq read names. The tokenizer is considerably smaller than general purpose tools on the more predictable name-sorted data. With chromosome and position sorted data, which scrambles the name ordering, the tokenizer is only beaten by the much slower ‘mcm’ tool.

**Table 3.** NovaSeq read name compression

	Name sorted			Position sorted		
Program	Size	Enc (MB/s)	Dec (MB/s)	Size	Enc (MB/s)	Dec (MB/s)
bzip2	2.52	32.08	222.11	6.18	15.97	137.88
gzip -12	2.14	3.78	250.59	5.50	2.80	241.93
bsc -m5e1tT	1.68	27.61	23.07	3.99	22.59	19.53
xz -9	1.31	2.50	74.98	4.87	1.70	65.31
mcm -m7	1.22	3.07	3.21	3.43	2.69	2.78
tok3 -3	1.11	24.68	49.61	3.55	22.98	67.84
tok3 -7	0.89	15.46	48.54	3.55	10.00	69.44
tok3 -19	0.88	8.82	41.59	3.48	4.84	39.13

*Note*: Performance of the read name tokenizer on name sorted and chromosome/position sorted NovaSeq reads. Size is shown in MB. Tok3 compression levels −3 and −7 use the rANS entropy encoder at low and medium tokenization levels, while −19 is the maximum compression level using adaptive arithmetic encoding. This uses the same htscodecs branch and test system as the rANS benchmark in Table 1.

Overall CRAM performance is not a single metric as it permits user-adjustable trade-offs between speed, size and granularity of random access. To a lesser extent this is true for BAM too with differing Deflate compression levels. We provide data for multiple format versions as well at several compression levels. The CRAM benchmarks are from the released version of SAMtools 1.13. Note, this does not yet include the SIMD-vectorized rANS entropy encoder and is using the 4-way Scalar implementation. Results using a vectorized build of SAMtools are presented in the Supplementary Material.

All decode timings are a read and complete decode of the files, with data discarded where possible. Encode timings are conversion from compressed BAM to a new file format. Full benchmarks and details are available in the Supplementary Material. The encoder and decoder were given 12 threads, using an Intel Xeon CPU E5-2660 running at 2.20 GHz. The quoted MPEG-G timings used 12 threads on an Intel Xeon E5-2670 at 2.6 GHz. Clock speeds for both systems are as reported by Intel, but may be subject to automatic boosting.

**Fig. 3.**
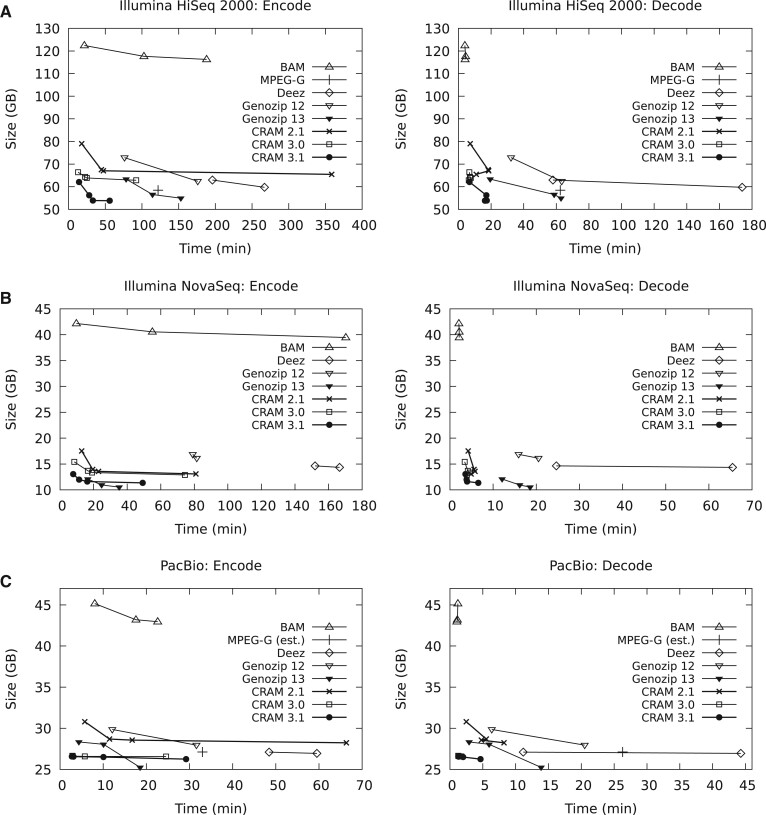
Benchmarks of aligned data formats using 12 threads. MPEG-G figures are taken from the Voges *et al.* paper, with ‘MPEG-G (est.)’ possibly using a slightly different input file (see text). Genozip 12 and Genozip 13 refer to versions 12.0.34 and 13.0.5, respectively, with the latter being released after the initial preprint publication and during manuscript review. Parts A, B and C show results for Human sample NA12878 sequenced using Illumina HiSeq2000, NovaSeq and PacBio CLR respectively.

Figure 3 shows the results for the Illumina HiSeq 2000 (ERR194147), Illumina NovaSeq (ERR3239334) and PacBio CLR (ftp://ftp.1000genomes.ebi.ac.uk/vol1/ftp/technical/working/20131209_na12878_pacbio/si/NA12878.pacbio.bwa-sw.20140202.bam). All three are Whole-Genome Shotgun libraries of NA12878.

Figure 3A shows the encode and decode speeds against file size for the Illumina HiSeq 2000 data (ERR194147). This is also MPEG-G dataset 02. Some formats have multiple points, linked together by a line. This represents different compression profiles used by the tools. In BAM, this adjusts the libdeflate compression levels to 6, 10 and the maximum of 12.

CRAM charts show profiles ‘normal’, ‘small’, ‘archive’ and ‘archive’ at compression level 9. The default ‘normal’ profile only uses the rANS codec and Deflate. The ‘small’ profile increases the block size and enables Bzip2 compression and in CRAM 3.1 the FQZComp codec. The ‘archive’ profile further increases block size, boosts the deflate compression level and in CRAM 3.1 enables the adaptive arithmetic coder. At compression level 8 and above the LZMA codec is also enabled for all CRAM versions. This has a significant cost to compression time so is often not a good speed/size trade-off, but is still fast at decoding.

It can be seen that CRAM 3.1 offers a similar leap over CRAM 3.0 compression ratios that it did in turn over CRAM 2.1. CRAM 3.1 is between 7% and 16% smaller than CRAM 3.0 at the equivalent profile, while being similar on speed except for ‘small’ and ‘archive’ decoding times. All CRAMs except those using LZMA encode faster than their BAM equivalents, while saving up to 56% storage, although BAM is quicker to decode. This is compatible with our design goal of being similar speed to BAM.

It is unclear whether the published MPEG-G benchmarks include auxiliary tags, but these only account for under 1% of this file. CRAM 3.1’s compression profiles straddle the MPEG-G file size, with the ‘small’ CRAM profile being both smaller and four times faster than MPEG-G. However, note the MPEG-G benchmarks were not performed by us, so there may be differences in measuring techniques.

Deez compression ratios are between CRAM 3.0 and CRAM 3.1 and a little behind MPEG-G. It is the slowest tool, although it should be noted that despite being given 12 threads it typically only utilized 2. Total CPU usage was less than MPEG-G. Genozip was also much slower than CRAM 3.1 while being larger, although during manuscript review updated versions of Genozip were released which are considerably closer on file size, with the most recent versions incorporating the CRAM codecs described here.

Figure 3B shows the same NA12878 sample sequenced using an Illumina NovaSeq instrument (ERR3239334). The improvements of CRAM over BAM here are more marked, due to the higher compressibility of the quantized quality values. The gains from CRAM 3.0 to CRAM 3.1 are also greater, with the ‘normal’ profile being 16% smaller. The CRAM 3.1 files are between 3.1 and 3.5 times smaller than BAM.

As before, Deez is slow and with this file does not match CRAM 3.0 for file size. No MPEG-G results are available for this dataset, but the published results for the NovaSeq MPEG-G dataset 37 implies good compression performance with a size similar to CRAM 3.1 archive mode. Genozip initially performed poorly, with file sizes larger than CRAM and considerably slower, while the recent revision gave superior compression ratios. The gain over CRAM 3.1 is almost entirely in the XA:Z auxiliary tags, which are abnormally large in this dataset. The latest Genozip releases now also integrate the new CRAM compression codecs described here.

Figure 3C shows an aligned PacBio CLR file, also for NA12878. This is the MPEG-G dataset 03. Published results are available for MPEG-G on this data, but we were only able to get our BAM and CRAM files to match their earlier published results (MPEG document M56361) by removing secondary alignments and discarding auxiliary tags. Hence, for comparison purposes, we applied these transformations to the downloaded BAM before performing this benchmark. We have been unable to verify with the authors if this is the correct procedure used in the MPEG-G publication, so it is listed here as ‘MPEG-G (est.)’.

This dataset shows a very minimal change between CRAM versions and compression profiles. The file is dominated by the quality values, which are largely uncompressible due to having a big range of discrete values (0–93) with very little correlation between successive values. Nevertheless, it is evident that CRAM saves a significant portion over BAM and CRAM 3.0/3.1 is a big improvement on the historic CRAM 2.1. The assumed MPEG-G size, Deez and Genozip 12 are all larger than CRAM 3.0 while being significantly slower. The most recent Genozip release has incorporated the quality encoding from ENANO (Dufort y Álvarez *et al.*, 2020), which utilizes sequence bases as a context for quality encoding. This has a significant reduction to PacBio size, but restrictives selective decoding of individual data types and has a significant CPU overhead. Nonetheless use of sequence as a context may be considered for future CRAM releases.

## 4 Discussion

CRAM has achieved the goals of providing a space-efficient alternative to BAM, while not suffering a significant time penalty. CRAM files in the European Nucleotide Archive significantly outnumber BAMs (personal communication) and it has seen wide adoption at many other sites. While some other tools are now smaller than CRAM 3.0 on some data files, this typically comes at a heavy cost in CPU. We demonstrate with CRAM 3.1 that changing just one component of the CRAM format, the compression codecs available, is sufficient for CRAM to remain competitive on size while not sacrificing its speed advantage. We also expect CRAM 3.1 to continue to improve as we learn how best to tune each codec, in particular the selection of the optimal FQZComp models.

However, there are wider format adjustments that could be made over and above adding new compression codecs, which will be addressed in CRAM 4.0. While an early draft of this already exists, CRAM 4.0 is likely to undergo further revisions. Improvements include migration of the rANS PACK and RLE filters to the CRAM slice encoding methods with additional transformations, such as integer delta encoding (useful for Oxford Nanopore Technology signal data) and an LZP step to help reduce repetitive auxiliary tags and to remove duplication in quality values due to secondary alignments. Further custom codecs may also need to be developed for the most expensive auxiliary tags, such as breaking down comma-separated tag formats (e.g. SA:Z and XA:Z) into components similar to the read name tokenizer. The general purpose compression interfaces used may also be reconsidered, such as replacing Deflate with Zstd and Bzip2 with Libbsc (Grebnov, 2011). There is scope for moving some compression meta-data, such as the Order-1 frequency tables in rANS, out of the data block produced by compression codecs and into the container header. This could permit more fine-grained random access with many slices per container sharing the same meta-data. Finally, improvements can be made to how embedded consensus sequences are handled, perhaps using a two-step delta as implemented in Deez.

It is evident however that some data does not gain compression with CRAM 3.1 due to the inherent randomness of the data held within, and no format improvement is likely to solve that. This is particularly true for some long-read technologies. We question the requirement to have so many distinct quality values in PacBio and ONT data and suggest lossy compression may be suitable for these datasets, prior to encoding in CRAM. We feel that this is best researched and addressed by the sequencing manufacturers and urge them to consider ways to reduce the data footprint of their output files.

An implementation of the CRAM 3.1 and 4.0 draft standards may be found in HTSlib (https://github.com/samtools/htslib). The individual codecs used are available as a separate library to permit use in other applications: HTScodecs (https://github.com/samtools/htscodecs).

## Supplementary Material

btac010_Supplementary_Data
