# Supplementary material for: CRAM 3.1: advances in the CRAM file format
Source: Bioinformatics. 2022 Jan 6;38(6):1497–503. doi: 10.1093/bioinformatics/btac010 (PMC8896640; doi:10.1093/bioinformatics/btac010)
Supplement: btac010_Supplementary_Data [file btac010_supplementary_data.pdf]

CRAM 3.1: Advances in the CRAM File Format
Supplementary Material

James Bonfield

December 14, 2021

Testing methodology

Encoding times were performed using SAMtools, Deez and Genozip converting from compressed BAM to the desired format. The decoding time of BAM is one of the fastest and is typically much faster than encoding time, so the impact of this on the results is low. Additionally we felt that compressed BAM offered the best format to read from as it reduced potential I/O bottlenecks, particularly when multi-threading.

During review new versions of Genozip were released (up to 13.0.5) with considerably improved compression ratios for some data sets. We retain the previous results, but show the improvements with the latest release too. Most noticeably it has improved compression of XA:Z auxiliary tags and at the best compression setting of PacBio quality values.

Decode times for SAMtools were performed using "samtools view -f 0xffff". This reads the data and filters out anything that doesn't have every bit-FLAG set, which is all reads. Hence there is no output and the time is dominated by decode only. Note for comparison against other tools, testing the fastest modes of CRAM decoding shows converting to uncompressed BAM adds 25% additional CPU cost over simply discarding the data. This further reduces in percentage with the slower CRAM profiles.

While we attempted the same filtering with Deez, it cleverly detected it could omit most of the work so the times were almost instantaneous. Therefore Deez decode timings unfortunately include outputting the data to stdout, which was then redirected to /dev/null to avoid file I/O costs. Similarly for Genozip we redirected output to /dev/null.

We do not know how the MPEG-G timings were performed other than the system they used (Intel Xeon E5-2670 at 2.6GHz).

Programs were given 12 threads for the aligned data and 8 threads for unaligned. The reason for difference is due to the availability of the MPEG-G benchmarks to compare against and we attempted to use a like for like test.

Times were measured using either "perf stat" or the unix "time" command, to record separately the user+system CPU time and Real (elapsed) time. The main paper shows the real time, but we also include CPU times below.

Unless specified otherwise, the machine used for benchmarking was an 16-core Intel Xeon CPU E5-2660 running at 2.20GHz with 2 hyper-threads on each core. The operating system was Ubuntu 18.04.5 LTS. Disk was fast networked file-systems; a mixture of Lustre and NetApp NFS. We were unable to clear the disk cache for these devices, but the tasks are mainly CPU bound so it is unlikely that disk I/O would become a significant bottleneck.

For CRAM, reference sequences were held as raw sequences (named after their md5sum) and fetched via NFS. Deez references were supplied as FASTA and Genozip used its own compressed reference format.

1 Tool version numbers

Deez: v1.9-beta2-26-g92cd56b (github clone)

SAMtools: 1.13 (linked against HTSlib 1.13 with libdeflate support).

HTScodecs: 1.1.1 Used internally by SAMtools/HTSlib and CRAM benchmarks.

HTScodecs: jkbonfield/unroll32 branch (9e9c3f7) used for SIMD tests.

MPEG-G: GenomSys encoding library 4.0 (personal communication).

Genie/GABAC: Develop branch (0382d5d)

Genozip: 12.0.34 and 13.0.5

SNAP: 1.0beta.24

1.1 Tool command lines

SAMtools: Encoding and decoding with SAMtools for BAM and CRAM tests are performed with

`samtools view -@12 -o -T` *ref.fa* `-O` *fmt,options* `-o` *out.fmt in.bam*

`samtools view -@12 -f 0xffff` *in.fmt*

Deez: Encoding and decoding with Deez is done via:

`deez -t 12 -!  -v1 -r` *ref.fa in.bam* `-o` *out.dz*

Genozip: Encoding and decoding with Genozip uses:

`genozip -e` *ref.genozip* `-@12 -f` *in.bam*

`genocat -e` *ref.genozip* `-@12 -z0 -o /dev/null` *in.bam.genozip*

We did not try the `--optimize` option as the manual states this is a lossy quality value compression.

Scripts to help with benchmarking can be found at https://github.com/jkbonfield/cram31_bench.

2 Exising CRAM 3.0 compression codecs

Current CRAM 3.0 has "external" library support via Deflate (bgzf / gzip), Bzip2 and LZMA. These follow the standard encoding formats published elsewhere.

CRAM also has it's own static frequency entropy encoder implemented with rANS, using either Order-0 or Order-1 statistics. This rANS implementation is a pure byte-wise entropy encoder with no data transformations (such as bit-packing or run-length encoding).

This is described in detail in section 14.4 of the CRAM 3.0 specification:

https://github.com/samtools/hts-specs/blob/master/CRAMv3.pdf

3 New CRAM 3.1 entropy encoders

CRAM 3.1 modifies the rANS entropy encoder normalisation to be 16-bits at a time for speed (CRAM 3.0 rANS is 8-bit) and adds internal support for bit-packing, run-length encoding and data-striping.

It also adds an alternative adaptive arithmetic encoder, as well as data-specific custom codecs for read name and quality value compression.

Demonstration JavaScript implementations of these codecs also exist in the HTScodecs package, although these should be consider as examples rather than production ready. They do however demonstrate the practicality of implementing CRAM 3.1 in a web environment, with JavaScript speeds typically 5x slower than their C counterpart.

The quality files used in testing here are available for download from ftp://ftp.sanger.ac.uk/pub/users/jkb/CRAM3.1/. Each of these are the first 1 million quality strings from a local NovaSeq (q4+dir) and a HiSeq 2000 run (q40+dir, from 9827_2#49.bam). The first column in each file is the quality string with the second column being a flag for first read (READ1) or second read (READ2). All entropy benchmarks were performed on the first column only with newlines removed, except for FQZComp which utilises the read start points and "selector" value.

3.1 Static 16-bit rANS coding

A modern CPU can dispatch multiple instructions per cycle and permits a degree of out-of-order execution. However there is a delay between starting an operation and the result being available. Hence a series of connected expressions (e.g. $b = a * 2; c = b + 17; d = c - a$) leads to stalling the instruction pipeline.

The existing CRAM 3.0 rANS entropy encoder interleaves 4 rANS states so independent calculations can be performed to avoid pipeline stalling. Each state renormalises 8-bits of data at a time (emitting 8-bits at a time during encoding). This is listed as "rANS4x8" below.

While CRAM 3.1 is still permitted to use the old rANS implementation, it adds a newer version which renormalises with 16-bits at a time for speed. Additionally it can select interleaving either 4 or 32 states. 4 is sufficient to avoid instruction pipelining for traditional scalar maths operations, but with 32 we can use 4 lanes of 8x32-bit SIMD vectors (AVX2) or 2 lanes of 16x32 (AVX512). Unfortunately the compilers are not able to auto-vectorise the code, but we have written multiple SIMD implementations in the HTScodecs github repository with the ability to detect the CPU and execute the fastest available. The CRAM 3.1 rANS codecs are listed as "rANS4x16" and "rANS32x16" below.

We present figures showing the encoding and decoding rates for various rANS options, with and without bit-packing and RLE, and with and varying degrees of vectorisation from none (compiler automatic, mostly traditional scalar code) to AVX512. As we needed AVX512 support, all Intel figures were tested on an Intel Xeon Gold 6242 CPU at 2.8GHz. We also present ARM Neon benchmarks on AWS Neoverse-N1 stepping r3p1.

Tables 1 and 2 show the performance of encoding NovaSeq quality values on Intel and ARM platforms. Huffman encoding is poor due to the need to emit at least 1 bit per symbol. Uncompressed data size is 151MB.

Table 3 shows the encoding of HiSeq 2000 quality values. As with the NovaSeq there is a considerable speed gain available by interleaving 32 rANS states and utilising SIMD, particularly when decoding. The uncompressed data file is 100MB.

In addition to the PACK and RLE methods, rANS also supports a STRIPE filter. This splits the data stream into N sub-streams consisting of every N^{th} byte, every $N^{th} + 1$ byte, and so on. Each of these are then compressed using their own rANS parameters. This can be particularly useful for BAM "B" auxiliary arrays. For example an "XX:B:i," auxiliary tag will store 32-bit numbers. If such numbers fall in the 1 to 100,000 range then bits 24-31 will always be 0 and bits 16-23 will be 0 or 1 only, with the bulk of the entropy in the first two bytes per value. STRIPE can permit RLE on the 4th byte, PACK on the 3rd byte, and Order-0 or Order-1 on the first two bytes.

Table 1: NovaSeq quality: entropy encoder speeds. Intel

Program	Option	Size(MB)	Enc(MB/s)	Dec(MB/s)
Zlib	Huffman	21.48	151	365
FSE	tANS	9.90	435	531
FSE	Huffman	20.74	710	1303
rANS4x8	O0	9.91	405	624
rANS4x16	O0	9.90	426	797
rANS32x16	O0	9.92	442	679
rANS32x16-SSE4	O0	9.92	445	1074
rANS32x16-AVX2	O0	9.92	709	1949
rANS32x16-AVX512	O0	9.92	847	2306
rANS4x8	O1	9.14	291	408
rANS4x16	O1	9.14	304	608
rANS32x16	O1	9.16	278	410
rANS32x16-SSE4	O1	9.16	281	693
rANS32x16-AVX2	O1	9.16	430	1484
rANS32x16-AVX512	O1	9.16	572	1762
rANS4x16	O0,RLE	10.51	419	862
rANS32x16-AVX512	O0,RLE	10.53	459	980
rANS4x16	O0,PACK	9.10	834	2176
rANS32x16-AVX512	O0,PACK	9.12	1051	3876
rANS4x16	O0,PACK+RLE	9.15	589	1195
rANS32x16-AVX512	O0,PACK+RLE	9.17	627	1302
rANS4x16	O1,RLE	8.74	394	820
rANS32x16-AVX512	O1,RLE	8.77	443	960
rANS4x16	O1,PACK	8.47	577	1463
rANS32x16-AVX512	O1,PACK	8.48	751	2344
rANS4x16	O1,PACK+RLE	8.24	488	987
rANS32x16-AVX512	O1,PACK+RLE	8.26	533	1078

Block size 1MB, except FSE Huffman which used 128KB. Compiler: Clang-13

Table 2: NovaSeq quality: entropy encoder speeds, on Arm NEON

Program	Option	Size(MB)	Enc(MB/s)	Dec(MB/s)
rANS4x8	O0	9.91	391.4	531.1
rANS4x16	O0	9.90	446.5	823.7
rANS32x16-Scalar	O0	9.92	476.9	675.3
rANS32x16-Neon	O0	9.92	476.6	1007.5
rANS4x8	O1	9.14	271.2	406.8
rANS4x16	O1	9.14	285.4	551.7
rANS32x16-Scalar	O1	9.16	265.4	354.0
rANS32x16-Neon	O1	9.16	264.2	675.4

3.2 Adaptive arithmetic coding

The adaptive arithmetic codec is best for non-stationary probabilities where the characteristics of the data may change throughout the block. However this is not a common feature of sequencing data, so the extra complexity is rarely required. This implementation of arithmetic coding is byte-

Table 3: HiSeq2000 quality: Entropy encoder speeds, on Intel

Program	Option	Size(MB)	Enc(MB/s)	Dec(MB/s)
Zlib	Huffman	50.65	116.3	257.2
FSE	tANS	50.12	439.9	596.1
FSE	Huffman	50.55	721.3	1206.3
rANS4x8	O0	50.13	361	614
rANS4x16	O0	50.12	425	797
rANS32x16	O0	50.13	384	681
rANS32x16-SSE4	O0	50.13	384	1078
rANS32x16-AVX2	O0	50.13	690	1910
rANS32x16-AVX512	O0	50.13	816	2247
rANS4x8	O1	48.45	271	342
rANS4x16	O1	48.43	286	560
rANS32x16	O1	48.44	253	401
rANS32x16-SSE4	O1	48.44	252	629
rANS32x16-AVX2	O1	48.44	390	1153
rANS32x16-AVX512	O1	48.44	517	1302

Block size 1MB, except FSE Huffman which used 128KB. Compiler: Clang-13

wise rather than the more usual bit-wise. This can be faster for low-order models, particularly on data sets with only a few symbols being used. As with the CRAM 3.1 rANS implementation, the arithmetic coder also includes the bit-packing, run length encoding and data-striping modes.

For comparisons with the other tools, Tables 4 and 5 show the the same NovaSeq and HiSeq2000 quality data along side a representative non-vectorised rANS line for comparison. Also included is MPEG-G's GABAC algorithm, as implemented in the Genie OpenSource tool. This is also an adaptive arithmetic codec (albeit bit-wise) with the ability to do bit-based transformations before entropy encoding, so is a good comparison against the arithmetic coder used in CRAM 3.1.

Note the Genie gabac-app tool requires little-endian 32-bit data, so the quality data was first transformed with a perl script. This transformation has not been included in the timing figures.

The adaptive arithmetic codec is an order of magnitude slower than rANS, but offers some modest improvement in ratios, particularly the NovaSeq data. GABAC is an order of magnitude slower again, but this is perhaps not indicative of the speed of a fully optimised implementation. It seems poor on compression ratio, but there may also be room for further improvement within the GABAC data format.

Table 4: HiSeq2000 quality: Entropy encoder speeds

Program	Option	Size(MB)	Enc(MB/s)	Dec(MB/s)
rANS4x16	O0	50.12	436.8	820.3
arith	O0	50.05	53.4	33.4
rANS4x16	O1	48.44	263.1	524.5
arith	O1	48.37	45.7	31.0
GABAC-app	-d15 -s1	50.10	3.6	5.5

Table 5: NovaSeq quality: entropy encoder speeds

Program	Option	Size(MB)	Enc(MB/s)	Dec(MB/s)
rANS4x16	O0,PACK+RLE	9.15	562.6	1247.1
arith	O0	9.83	120.0	94.5
arith	O0,PACK+RLE	9.16	220.0	188.3
rANS4x16	O1,PACK+RLE	8.24	464.4	1001.9
arith	O1	9.12	105.3	91.3
arith	O1,PACK+RLE	8.12	156.5	115.1
GABAC-app	-d15 -s1	8.40	5.0	8.0

3.3 Generalised FQZComp

The original FQZComp tool had a choice of three quality models, selected by parameters -q1, -q2 and -q3 using 12-bits, 16-bits and 20-bits respectively for the model context. The construction of these contexts were hard coded.

CRAM 3.1's FQZComp model is designed around block-based compression offering random access, so uses at most 16-bits of context. The construction of this context is configurable with the construction rules stored in the data stream so the decoder can follow the instructions during decode. This offers considerable freedom in context construction.

For example, the base qualities on long read technologies may not vary much along the length of the read, so attempting to utilise position on the sequence will likely harm compression by making the model slower to adapt. Conversely Illumina data has a strong bias between the 5' and 3' end. Additionally this also means knowing the orientation can help improve compression.

CRAM's codecs are designed to be operated in isolation without knowledge of other CRAM fields. This is a deliberate design decision in order to remove data dependencies and permit a more column-based filtering access pattern. However the additional bit required per read to store (duplicate) the orientation within the quality stream may be more than offset by space saving. It also permits a flag per read to indicate entire quality string duplication, which is beneficial when supplementary or secondary alignments are present.

Other data available for use in context construction are previous quality values, a running total of successive quality differences since the start of this read, and the position along the current read. These values can be indirected via a lookup table, so they do not need to be uniformly distributed.

We can also store part of the context from the data stream itself, stored at the start of each new record. We use this to classify data by a variety of methods. For example separating by READ1 and READ2 flags can sometimes be beneficial. We may also wish to group reads by their average quality value, the number of differences, or even their position on the flow cell (extracted from the read name) with edge-effects or bubbles changing quality characteristics. Any categorisation can be considered by the encoder as the decoder simply uses data from the file format without knowing how it was derived. These are known as the "selector" bits.

Pseudo-code for context construction used during the decoding process is below. See CRAM-codecs specification for more details.

Table 6 shows the performance of FQZComp on the HiSeq 2000 and NovaSeq test sets, with several different context configurations. Also shown is libbsc, as a high quality commonly used general purpose compression tool having a similar data throughput, and the original 2012 standalone FQZComp tool. We also repeat the best rANS and arith ratios for ease of comparison. It is clear that the generalised CRAM implementation of the FQZComp quality model is a significant advance over the original, although this general purpose nature currently has a small speed penalty.

```
function FQZUpdateContext(params, q)
    ctx ← params.context                                  ▷ Also the initial value
    qctx ← (qctx << params.qshift) + qtab_q
    ctx ← ctx + ((qctx AND (2^params.qbits − 1)) << params.qloc)
    if params.pflags AND 32 then                          ▷ have_ptab
        p ← Min(pos, 1023)
        ctx ← ctx + (ptab_p << params.ploc)
    end if
    if params.pflags AND 64 then                          ▷ have_dtab
        d ← Min(delta, 255)
        ctx ← ctx + (dtab_d << params.dloc)
        if prevq ≠ q then
            delta ← delta + 1
        end if
        prevq ← q
    end if
    if params.pflags AND 8 then                           ▷ do_sel
        ctx ← ctx + (sel << params.sloc)
    end if
    return ctx AND (2^16 − 1)
end function
```

Table 6: Quality value FQZComp performance

Program	Option	Size(MB)	Enc(MB/s)	Dec(MB/s)
NovaSeq qualities				
rANS4x16	O1,PACK+RLE	8.24	464.4	1001.9
arith	O1,PACK+RLE	8.12	156.5	115.1
bsc-3.1.7	-m0e2tTp	7.72	19.3	35.8
FQZComp-4.6	-q2	8.12	46.2	40.8
FQZComp-4.6	-q3	7.72	41.9	37.8
CRAM FQZComp	-s0	7.27	28.6	51.8
CRAM FQZComp	-s1	7.29	26.9	45.7
CRAM FQZComp	-s1+read 1/2	7.21	21.4	26.7
HiSeq 2000 qualities				
rANS4x16	O1	48.4	263.1	524.5
arith	O1	48.4	45.7	31.0
bsc-3.1.7	-m0e2tTp	42.6	6.5	9.4
FQZComp-4.6	-q2	44.9	23.3	20.0
FQZComp-4.6	-q3	41.0	19.6	16.7
CRAM FQZComp	-s0	42.5	15.8	17.8
CRAM FQZComp	-s1	34.0	16.4	18.8
CRAM FQZComp	-s1+read 1/2	31.3	13.3	14.9

Compression of NovaSeq and HiSeq 2000 quality values using libbsc and CRAM 3.1's FQZComp and the original FQZComp-4.6 algorithm, in 10 blocks of 100,000 records. Tested on an Intel Xeon Gold 6242 CPU at 2.8GHz

3.4 Name tokeniser

The name tokeniser deconstructs a read name into a series of elements and then compares each element against a previous tokenised read name. Typically this is the immediately previous name, but with mixed data sets it may be an earlier one matching the same pattern. These tokens are then collated in columns with each column then compressed using either the CRAM 3.1 rANS or adaptive arithmetic coders.

Table 7 shows the compression performance on position-sorted and name-sorted data files. We show results for our name tokeniser (tok3) against brotli (bro), libbsc (bsc), bzip2, gzip (libdeflate implementation), mcm, xz and zstd. Tok3 compression levels 1 to 9 use rANS encoding and 11 to 19 use adaptive arithmetic coding. The last digit governs how hard it searches to find the optimal encoding parameters.

The position sorted data is the aligned NovaSeq file used in the CRAM benchmarks (ERR3239334). The read name sorted data set is the interleaved read1/read2 records from ERR174326. Both data sets are 1 million names, separated into 10 lots of 100,000 names to simulate coarse random access capabilities.

Mcm and libbsc are strong contenders, with mcm sometimes ahead of the CRAM name tokeniser on ratio, however as with other codecs CRAM aims to strike the balance between speed and size.

Table 7: Name / identifier compression

	Name sorted			Position sorted		
Program	Size(MB)	Enc(MB/s)	Dec(MB/s)	Size(MB)	Enc(MB/s)	Dec(MB/s)
tok3 -3	1.11	24.68	49.61	3.55	22.98	67.84
tok3 -7	0.89	15.46	48.54	3.55	10.00	69.44
tok3 -19	0.88	8.82	41.59	3.48	4.84	39.13
bro -3	2.90	90.49	164.71	7.03	67.13	176.46
bro -9	2.79	9.68	177.69	5.91	6.28	203.53
bro -11	1.51	0.47	164.71	5.01	0.36	183.18
bsc -m0e2tT	2.60	8.27	29.29	4.06	7.50	28.00
bsc -m5e1tT	1.68	27.61	23.07	3.99	22.59	19.53
bzip2	2.52	32.08	222.11	6.18	15.97	137.88
gzip -6	2.87	75.76	254.95	6.58	54.95	240.42
gzip -12	2.14	3.78	250.59	5.50	2.80	241.93
mcm -t1	1.42	5.31	5.57	3.59	4.73	4.80
mcm -m7	1.22	3.07	3.21	3.43	2.69	2.78
xz -1	2.15	27.32	78.81	6.53	15.84	61.06
xz -9	1.31	2.50	74.98	4.87	1.70	65.31
zstd -9	2.51	39.73	229.05	6.22	21.49	236.00
zstd -19	1.56	2.61	225.53	5.02	1.81	224.96

4 HTSlib's CRAM implementation

The CRAM specification describes the byte stream and how it must be decoded. However there are many possible byte streams that decode to the same data and hence considerable freedom is available to the encoder. We can trivially see similar choices in general purpose tools such as gzip, where the user can select compression methods -1 to -9 governing the algorithms used by the encoder (and how much time they spend), but all decoders can process the compressed file.

CRAM extends on this further by permitting different choice of algorithms as well as their own internal compression levels, for example selecting from bzip2, gzip or rANS.

Some of these choices are controllable by the user, while others are automatically chosen. User controllable options:

- A general compression level, indicating the trade-off between CPU time and file size.
- The choice of which compression codecs may be used. This does not mean they will be used for all data blocks, but if a compression codec offers a significant advantage then it may be selected.
- The number of records and/or bases per slice, and the number of slices per container. This governs the random access capabilities of the file format. Note HTSlib's number of sequences per slice has a nominal cap of 500bp per sequence unless explicitly changed. This avoids the presence of long reads from producing excessively large containers.
- Whether to encode against an external reference sequence, an internal reference sequence, or referenceless.
- A compression profile. This is a simple way of specifying the above options as a single parameter. The profiles available are:

  fast
  Sets compress level to 1 and disables the name tokeniser. 10,000 sequences per slice.

  normal
  The default compression level (5). Name tokeniser is enabled and there are 10,000 sequences per slice.

  small
  Compression level is set to 6 unless otherwise changed. Bzip2 and fqzcomp quality modes are enabled and 25,000 sequences per slice.

  archive
  Compression level is set to 6 unless otherwise changed. Bzip2, fqzcomp quality modes and the adaptive arithmetic coder are enabled (both as a standalone codec and also as part of the name tokeniser). If compression level was previously set to 7 or above then Lzma is also enabled. The number of sequences per slice is set to 100,000. Use of lzma is typically the main source of difference between "archive" and "archive9" in the tables below.

HTSlib's CRAM implementation was derived from the Staden io_lib package, and both share a similar strategy. The encoding process is as follows.

1. Read sufficient records to fill a container, and produce the uncompressed CRAM data series associated with these records. New containers are also started when a reference changes, unless this leads to many small containers in which case multi-reference containers are used with an additional data-series present to hold the reference ID.

   Data-series are analogous to SAM columns, such as identifier, chromosome, position, qualities. Sequence is split into multiple data series based on the delta to the reference (such as length of deletion, SNPs, etc). Each auxiliary tag type is also assigned its own data series.

Modern CRAM implementations store each data series in their own block, but earlier versions would merge some together. Merging may boost compression where correlation between values occur, but it reduces the ability to do efficient data-type selective retrieval.

2. Gather statistics on each data series, such as the number of distinct values for an integer series and their minimum and maximum values. Data series that have constant values may be encoded using a Huffman tree with a zero-length bit stream (ie they consume no space other than the Huffman meta-data). In CRAM 4 this is achieved with a special CONST encoding type.

   CRAM 4 also adds a new variable sized integer encoding which can distinguish signed vs unsigned values and uses the minimum value as a baseline to compare against.

3. For all other data blocks, a list of candidate compression codec methods is maintained. This is initially everything permitted by the user (not including bzip2 or lzma unless explicitly requested for CRAM 3.0, and arithmetic coder and fqzcomp quality encoder for CRAM 3.1).

   Note that a single compression codec listed in the CRAM specification may be listed as multiple methods, as a codec may have several possible choices. For Gzip this consists of GZIP-RLE (run length encoding only), GZIP-1 (level 1 only), and GZIP (at the current compression level). FQZComp has 4 different sets of parameters to trial different encoding models. CRAM 3.0's 8-bit rANS has Order-0 and Order-1 encodings. CRAM 3.1's 16-bit rANS has Order-0, Order-1, plus combinatons including run-length encoding and bit-packing. Similarly for the CRAM 3.1 arithmetic coder. This gives a large choice of available methods.

   Compression level can restrict this initial choice.

   Level 1 removes gzip, bzip2 and lzma support for quality scores (but may still use gzip in run-length-encoding mode). No rANS RLE and bit-packing are permitted.

   Level 2 permits standard gzip for all data series, but still only uses gzip level 1 for quality scores and bases. Some combinations of rANS bit-packing and RLE are enabled.

   Levels 3 and above permit the external codecs (gzip, bzip2, lzma), if enabled, at the compression level selected.

   Levels 6 and above also enable further rANS bit-packing and RLE combinations.

   The data series also governs which initial methods to utilise. If Fqzcomp is enabled, quality scores are compressed using 1 pre-defined parameter set at levels 1-4, two parameter sets at levels 4 and 5, and 4 parameter sets at levels 6 and above.

   The read identifier data series removes the rANS methods and GZIP-RLE from the initial method list as they are almost never beneficial.

4. Each data series is then compressed by trialling each of the available methods listed for that specific data series type. A cost function is applied based on an estimated decoding speed and the compression level. At level 9 speed is ignored, while at level 1 it is has a high consideration. Thus if a compression method yields only 1% smaller data but is likely 100% slower then it may not be utilised for encoding.

   The output size of each tested method is summed per data series.

5. After a fixed (low) number of trials, a decision is made on which method is best and it is then applied without trial for the next (high) number of containers.

6. This trial and deploy strategy is repeated multiple times. A secondary level of trial statistics are maintained so if a data series is consistently rejected by a considerable margin then it will be dropped from subsequent trial phases.

   This way HTSlib learns the nature of the data and which methods work best. This is particularly important for auxiliary data which may contain any data with unknown characteristics. Hence speed assessment of HTSlib on small files may not be indicative of speed assessments on larger ones.

7. Specific events which may change the nature of the data, such as moving from mapped to unmapped or many large references to many small ALT references, can trigger a reset of the learnt compression metrics.

Note that this learning strategy is specific to HTSlib and the Io_lib code it was derived from. The HTSJDK implementation has fixed compression methods for each data series. This may give a speed advantage on small files, but it lacks the ability to adapt to the data.

This is one example of where evaluation of the CRAM file format must be distinguished from evaluation of specific implementation methods. However we cannot evaluate the format without evaluating at least one implementation. How best to select the encoding methods and compression codecs per data-series is an open question, however we choose to evaluate HTSlib here as we believe it to be the best of the currently available implementations, but there is still potential for future improvements while retaining CRAM compatibility.

5 Detailed CRAM file compression results: aligned data

The "io_lib" `cram_dump` tool can be used to interrogate the layout of a CRAM file. An example container header is listed below.

```
Container_header block pos 4628
  Preservation map:
    RN => 1 (0x1)
    SM => 94095715340138 (0x55945db00f6a)
    TD => 94095715340100 (0x55945db00f44)
    AP => 0 (0x0)
  Substitution map:
    A: CGTN
    C: AGTN
    G: ACTN
    T: ACGN
    N: ACGT
  TD map:
      0: PLZPUZLBZSMZPGZ
      1: PLZPUZLBZSMZPGZNMC

  Record encoding map:
     FN =>         EXTERNAL {26}
     FP =>         EXTERNAL {28}
     RL =>          HUFFMAN {1, 101, 1, 0}
     RN =>  BYTE_ARRAY_STOP {0, 11}
     QS =>         EXTERNAL {12}
     BA =>         EXTERNAL {30}
     BB =>   BYTE_ARRAY_LEN {1, 1, 42, 1, 1, 37}
     TL =>         EXTERNAL {32}
     IN =>  BYTE_ARRAY_STOP {0, 13}
     BF =>         EXTERNAL {15}
     MF =>          HUFFMAN {1, 0, 1, 0}
     TS =>          HUFFMAN {1, 0, 1, 0}
     CF =>          HUFFMAN {1, 3, 1, 0}
     AP =>             BETA {0, 28}
     FC =>         EXTERNAL {27}
     MQ =>          HUFFMAN {1, 0, 1, 0}
     DL =>         EXTERNAL {29}
     BS =>         EXTERNAL {31}
     NP =>          HUFFMAN {1, 0, 1, 0}
     SC =>  BYTE_ARRAY_STOP {0, 14}
     NS =>          HUFFMAN {1, 255, 255, 255, 255, 15, 1, 0}
     RG =>          HUFFMAN {1, 0, 1, 0}
     RI =>         EXTERNAL {33}

  Tag encoding map:
    PUZ =>  BYTE_ARRAY_STOP {9, 224, 80, 85, 90}
    LBZ =>  BYTE_ARRAY_STOP {9, 224, 76, 66, 90}
    SMZ =>  BYTE_ARRAY_STOP {9, 224, 83, 77, 90}
    NMC =>   BYTE_ARRAY_LEN {3, 4, 1, 1, 1, 0, 1, 4, 224, 78, 77, 67}
    PGZ =>  BYTE_ARRAY_STOP {9, 224, 80, 71, 90}
    PLZ =>  BYTE_ARRAY_STOP {9, 224, 80, 76, 90}
```

The record encoding map describes how each data series is encoded. This is mostly outputting to blocks as-is (EXTERNAL) or with variable length data (either null terminated or with explicit length encodings). HUFFMAN is also (ab)used in this example for constant items, with a Huffman tree containing a single zero-bit long symbol. The BETA encoding is also used for alignment positions. The parameters to each encoding describe, among other things, the block it is stored in, for example soft-clipped sequence ("SC") is written to block number 14.

The blocks are then grouped together in slices, and each block describes the compression codec used for that data type.

```
Slice 1/1, container offset 294, file offset 4922
    Slice content type MAPPED_SLICE
    Slice ref seq    -2
    Slice ref start  0
    Slice ref span   0
    Slice MD5        00000000000000000000000000000000
    Rec counter      0
    No. records      25000
    No. blocks       20
    Blk IDS:         {11, 12, 13, 14, 15, 26, 27, 28, 29, 30, 31, 32, 33, 5261146, 5131587, 5264730, 5459290, 4997722, 5262426}
    Ref base id:     -1
```

```
Block 1/20
    Size:         86843 comp / 87500 uncomp
    Method:       GZIP  (1)
    Content type: CORE
    Content id:   0

Block 2/20
    Size:         24561 comp / 746680 uncomp
    Method:       TOK3_R        (8)
    Content type: EXTERNAL
    Content id:   11
    Keys:         RN

Block 3/20
    Size:         676771 comp / 2525000 uncomp
    Method:       FQZ   (7)
    Content type: EXTERNAL
    Content id:   12
    Keys:         QS
```

The “io_lib” `cram_size tool` can summarise all this data, reporting how much space each data series consumes and which compression methods are used (this may be more than one as a different codecs may be used in different slices).

```
Block CORE              , total size  1439857150
Block content_id      11, total size   403313878               n            RN
Block content_id      12, total size 12771259069              f             QS
Block content_id      13, total size     2632067      01                    IN
Block content_id      14, total size   194147009       1  8                 SC
Block content_id      15, total size    50899663       1  8                 BF
Block content_id      26, total size    80976004      01                    FN
Block content_id      27, total size    38717234      01  8                 FC
Block content_id      28, total size   219404316 g    0                     FP
Block content_id      29, total size     1517445      0                     DL
Block content_id      30, total size   282950932       1   9                BA
Block content_id      31, total size    47329198      014                   BS
Block content_id      32, total size     9824542      01                    TL
Block content_id      33, total size   185392546  b      5                  RI
Block content_id 4997722, total size      332138            2               LBZ
Block content_id 5131587, total size     3146066      0                     NMC
Block content_id 5261146, total size     4350689 g         9                PGZ
Block content_id 5262426, total size     1228921           9                PLZ
Block content_id 5264730, total size      332138            2               PUZ
Block content_id 5459290, total size      332256 g          2               SMZ
```

In the tables below, we report total file size and time taken to encode and decode, gathered using the `time` command. This shows the difference between total CPU usage across all threads and the elapsed time, giving an indication of thread scalability. The entry for “Deez q1” corresponds to the `-q1` option of Deez which uses the samcomp quality model. Random access is not available on the quality field for this method, so it is excluded from the main figure.

We also include numbers for the experimental CRAM 4.0 version. This is ongoing work and is expected to change further, but the primary reason for size reduction over 3.1 is deduplication of read names and the option to store quality values in their original 5’ to 3’ orientation.

Figure 1 is a zoomed up colourised version of the primary figure in the paper, showing more details on the relative performance of the different CRAM versions. Because of the zooming, BAM is off the top of the chart and Deez’s poor multi-threading scaling mostly hides the results to the right. Figure 2 is the same data showing the CPU time (User + System). This now includes Deez, which is quite performant. MPEG-G is not shown as we only have the elapsed time for that test system.

The improvements with Genozip in the 12.0.34 to 13.0.5 upgrade are particularly effective for the NovaSeq data, giving the smallest file from all tools tested. The primary gain over CRAM is improved compression of the XA:Z auxiliary tag, which consumes over 20% of that data set in CRAM.

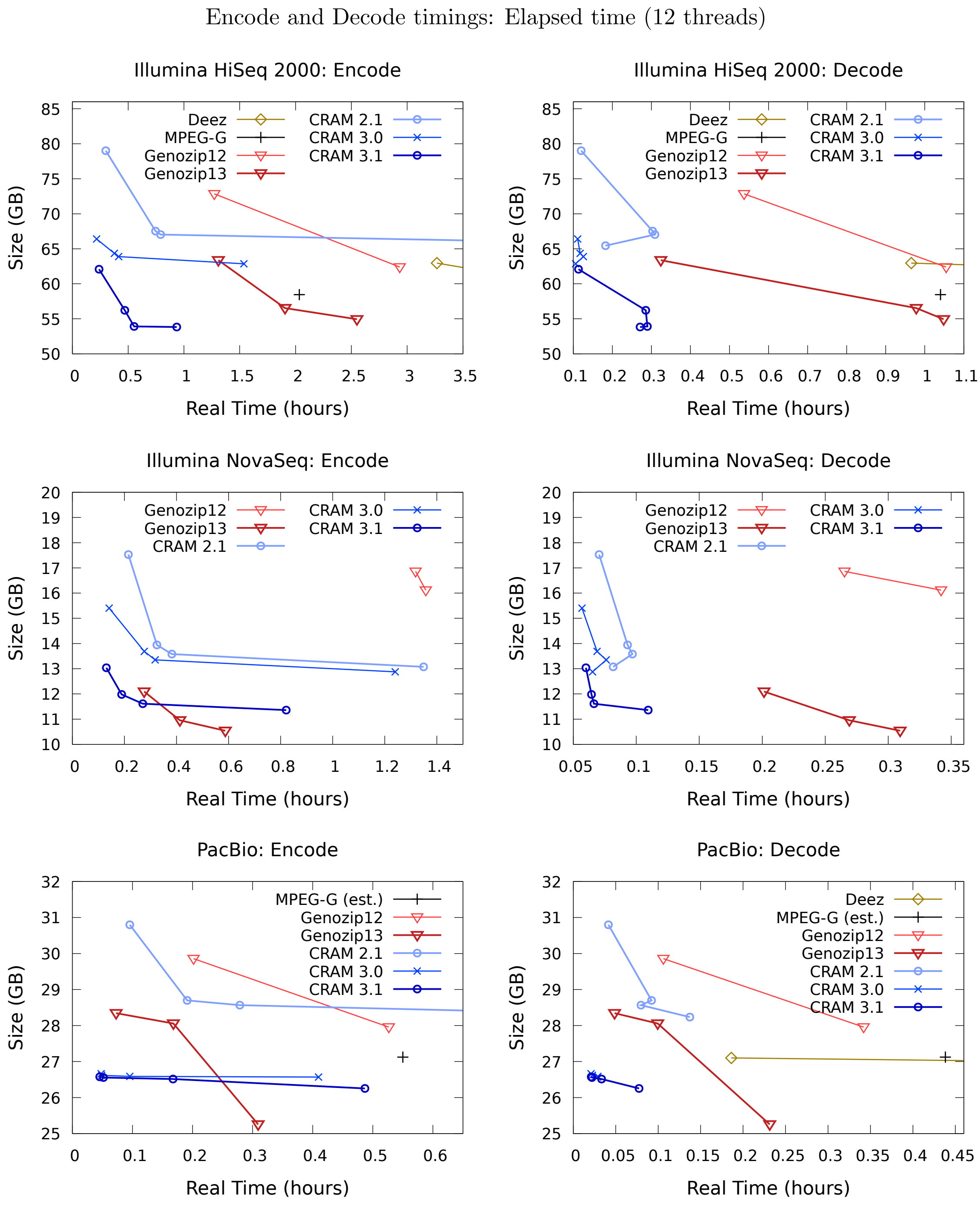

Figure 1: Multi-threaded benchmark of aligned data formats showing the elapsed time. MPEG-G figures are taken from the Voges et.al paper, with "MPEG-G (est.)" assumed to be working on the same data file as our CRAM measurements, but this is not certain. (See text.) Deez is absent as the elapsed time is too large. BAM is not shown as the file sizes are too big. Genozip12 and Genozip13 refer to versions 12.0.34 and 13.0.5 respectively.

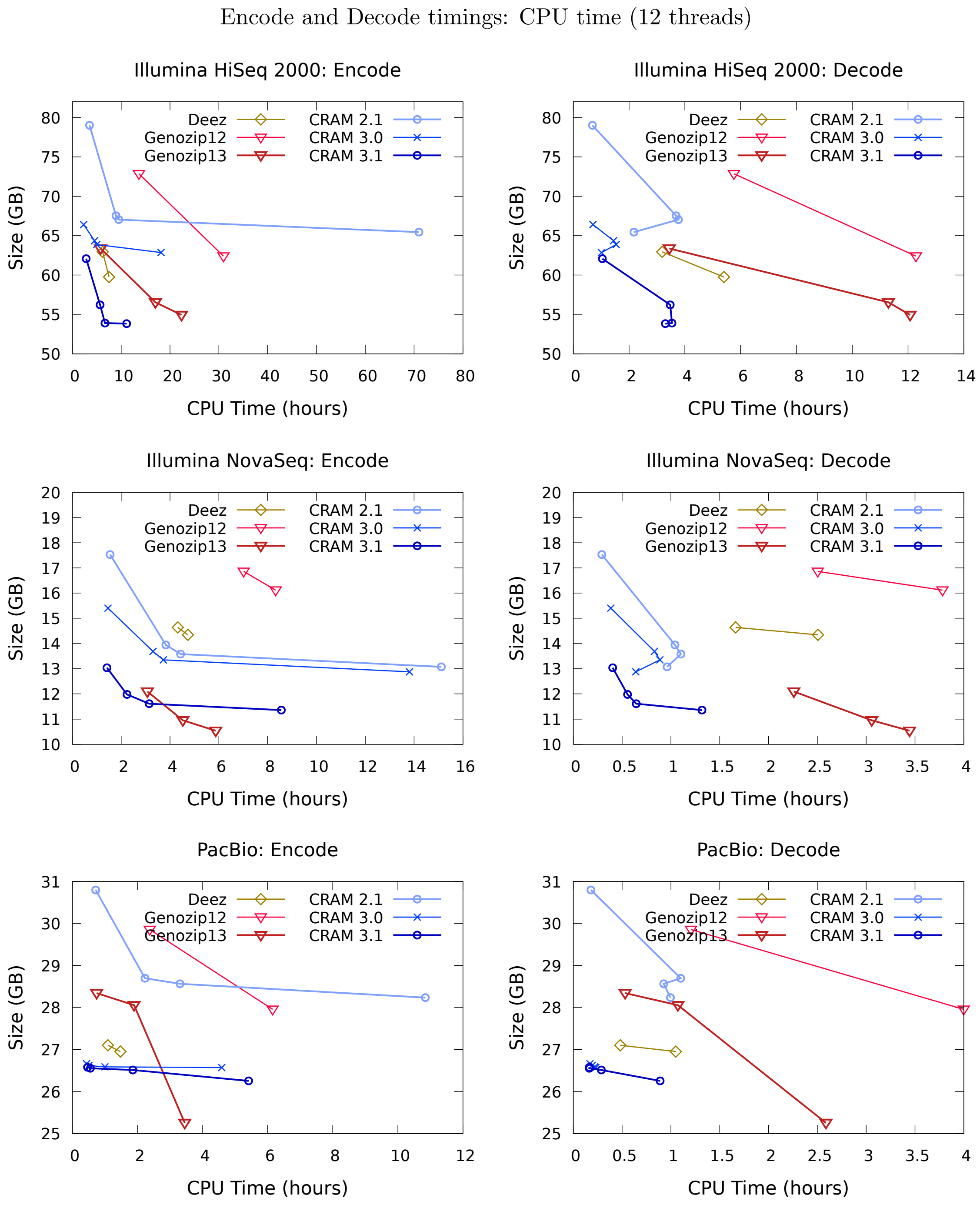

Figure 2: Multi-threaded benchmarks of aligned data formats showing the CPU (User + System) time. MPEG-G figures are taken from the Voges et.al paper, with "MPEG-G (est.)" assumed to be working on the same data file as our CRAM measurements, but this is not certain. (See text.) BAM is not shown as the file sizes are too big. MPEG-G is absent as only elapsed time was reported. Genozip12 and Genozip13 refer to versions 12.0.34 and 13.0.5 respectively.

5.1 NovaSeq data

We tried to obtain details on how the MPEG-G dataset 37 was aigned, in order to compare tools against MPEG-G, but were unable to obtain them or the BAM file. Hence the results below use a difference public data set.

Source: ftp://ftp.sra.ebi.ac.uk/vol1/run/ERR323/ERR3239334/NA12878.final.cram

Figure 3 shows the break down in data type sizes for CRAM 2.1 (normal and small profiles), CRAM 3.0 (normal, small and archive level 9), CRAM 3.1 (normal, small and archive level 9), CRAM 4.0 (normal and small), Deez (–q1 and –q2) and Genozip (normal and –fast). Sequence size is everything that is not Name, Qual or Auxiliary fields and hence also includes mapping quality, template lengths and flags. This is to make it easier to compare between tools.

Deez is significantly ahead on sequence here, presumably due to the two-level delta, while the new CRAM codecs are significantly ahead on read names and quality values.

The updates that occurred in Genozip during the review of this manuscript are most noticable on this data set. The sequence encoding is considerably improved and the auxiliary tag encoding (dominated by the large "XA:Z" tag) is now the best of any tool. This backs up our discussion point in the main paper over tag specific encoding. Most of this size reduction occurred in 13.0.5, but since Genozip 13 the CRAM codecs presented here have also been incorporated for some improved speed and further ratio gains.

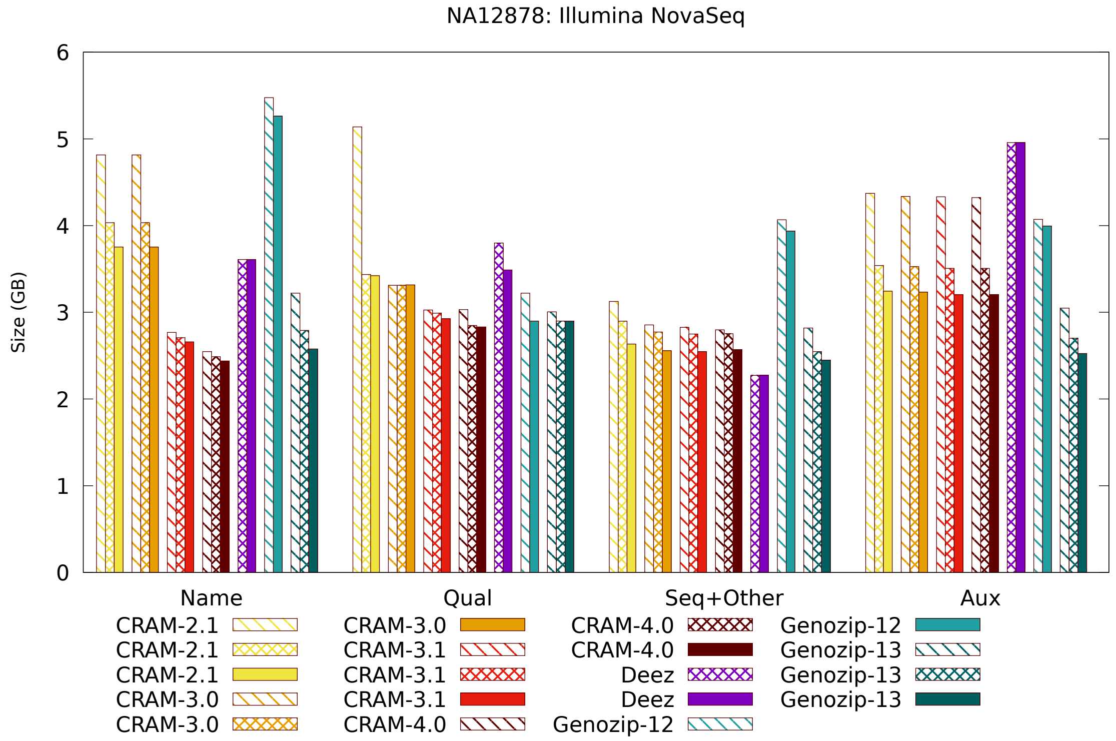

Figure 3: A break down of the relative size for each data type. Successive bars for the same format represent more advanced compression options. Genozip-12 and Genozip-13 refer to versions 12.0.34 and 13.0.5 respectively.

Memory usage is also shown in Table 8, but note with 12 threads each tool will have 12 times as much data in memory, with associated compression buffers and statistics. Single threaded conversions will use substantially less RAM. As we switch between CRAM profiles the memory also changes, which is primarily down to increasing the number of sequences per slice (the unit of random access). BAM is by far the lowest memory user, due to having a very small 64k

compression buffer as required by bgzf.

Table 8: NovaSeq detailed compression timings

Format	Size (MB)	Encode time(s) / Mem(MiB) CPU	Elapsed	Peak Mem	Decode time(s) / Mem(MiB) CPU	Elapsed	Peak Mem
BAM normal	42,158	5,124	583	27	767	125	14
BAM small	40,552	39,044	3,299	47	766	128	14
BAM archive	39,458	122,968	10,216	48	767	129	14
CRAM 2.1 normal	17,526	5,552	774	2,549	1,050	254	673
CRAM 2.1 small	13,944	13,779	1,169	4,832	3,750	335	737
CRAM 2.1 archive	13,578	15,959	1,376	12,423	3,966	349	2,253
CRAM 2.1 archive9	13,071	54,420	4,857	15,272	3,460	294	2,722
CRAM 3.0 normal	15,404	5,247	509	1,891	1,384	205	630
CRAM 3.0 small	13,686	11,888	994	4,764	2,995	248	793
CRAM 3.0 archive	13,376	13,413	1,145	12,126	3,194	274	2,432
CRAM 3.0 archive9	12,879	49,712	4,464	15,316	2,304	235	3,246
CRAM 3.1 normal	13,039	5,101	470	2,358	1,453	216	806
CRAM 3.1 small	11,994	8,058	682	5,993	2,001	232	2,109
CRAM 3.1 archive	11,625	11,319	973	15,995	2,321	239	5,361
CRAM 3.1 archive9	11,355	30,812	2,956	18,656	4,744	394	4,574
CRAM 4.0 normal	12,789	4,911	464	1,948	1,431	232	815
CRAM 4.0 small	11,637	11,828	784	6,123	4,586	373	1,949
CRAM 4.0 archive	11,262	14,333	1,209	16,101	4,750	395	4,788
CRAM 4.0 archive9	11,056	29,072	2,778	18,720	4,656	381	4,531
Deez (normal)	14,641	15,552	9,119	3,909	5,984	1,479	4,236
Deez q2	14,342	17,039	9,994	3,839	9,022	3,929	5,264
Deez q1	13,840	17,426	10,314	4,273	9,736	4,505	4,666
Genozip 12.0.34 fast	16,836	25,267	4,746	6,656	9,012	955	1,803
Genozip 12.0.34 (normal)	16,115	29,977	4,887	6,587	13,613	1,232	2,249
Genozip 13.0.5 fast	12,096	11,062	993	3,864	8,130	725	3,431
Genozip 13.0.5 (normal)	10,954	16,299	1,486	3,984	11,004	969	3,390
Genozip 13.0.5 best	10,537	21,143	2,114	23,998	12,397	1,114	16,643

Note: Deez -q1 lacks random access for quality values.

5.2 HiSeq 2000 data

This is the Illumina Platinum Genomes deep sequencing of NA12878. It is also the MPEG-G Data-Set 02.

Source: ftp://ftp.sra.ebi.ac.uk/vol1/run/ERR194/ERR194147/NA12878_S1.bam

The MPEG-G format quoted size from their paper supplementary material is 58444073745 bytes, at 8,280s to encode and 3,990s to decode using 8 threads on an Intel i7-7700 running at 3.6Hz. The earlier MPEG document M56361 quotes the size as a very similar 58457106765, taking 7,320s to encode and 3,745s to decode using 12 threads on an Intel E5-2670 running at 2.6GHz. Our tests are on an Intel E5-2660, also with 12 threads, nominally running at 2.2GHz but with CPU frequency scaling potentially overclocking this. We believe the speed benchmarks to be sufficiently similar to be of value in comparisons, although we cannot quantify the impact of any I/O speed differences.

Figure 4 shows the break down in data type sizes for CRAM 2.1 (normal and small profiles), CRAM 3.0 (normal, small and archive level 9), CRAM 3.1 (normal, small and archive level 9), Deez (–q1 and –q2), MPEG-G and Genozip (normal and –fast). Sequence size is everything that is not Name, Qual or Auxiliary fields and hence also includes mapping quality, template lengths and flags. This is to make it easier to compare between tools.

The quality values dominate the file size here, giving CRAM 3.1 a significant advantage in the overall file size. However the new name takeniser is clearly a visible gain too.

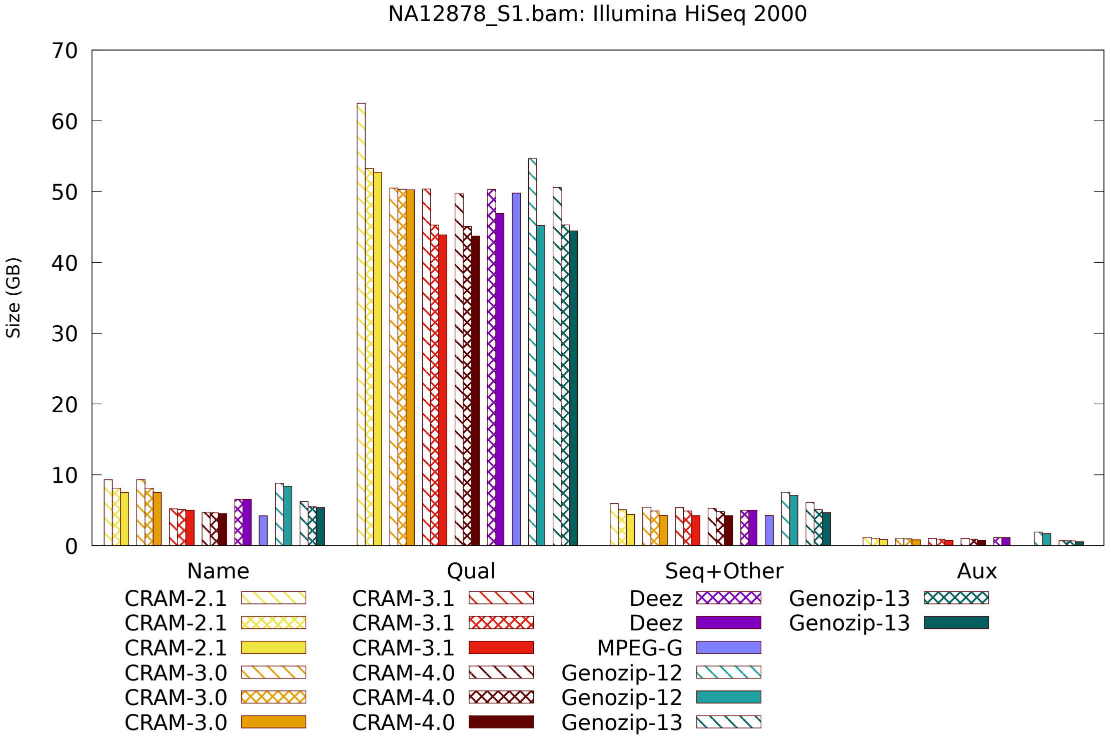

Figure 4: A break down of the relative size for each data type. Successive bars for the same format represent more advanced compression options.

Table 9: HiSeq 2000 detailed compression timings

Format	Size (MB)	Encode time(s) / Mem(MiB) CPU	Encode Elapsed	Encode Peak Mem	Decode time(s) / Mem(MiB) CPU	Decode Elapsed	Decode Peak Mem
BAM normal	122,405	11,238	1,293	25	1,598	232	12
BAM small	117,644	71,922	6,165	44	1,599	264	12
BAM archive	116,251	135,301	11,286	46	1,621	245	13
CRAM 2.1 normal	79,011	12,646	1,076	1,053	2,468	433	840
CRAM 2.1 small	67,518	32,116	2,685	1,360	13,275	1,091	672
CRAM 2.1 archive	67,028	34,206	2,846	2,484	13,577	1,113	1,402
CRAM 2.1 archive9	65,445	255,628	21,512	3,539	7,826	657	1,627
CRAM 3.0 normal	66,412	8,294	781	1,109	2,539	401	887
CRAM 3.0 small	64,358	16,333	1,354	1,426	5,197	423	972
CRAM 3.0 archive	63,877	17,975	1,492	2,536	5,540	453	1,878
CRAM 3.0 archive9	62,859	65,375	5,533	3,878	3,634	381	2,493
CRAM 3.1 normal	62,063	10,142	859	1,290	3,760	408	977
CRAM 3.1 small	56,219	20,420	1,692	2,606	12,513	1,028	2,039
CRAM 3.1 archive	53,909	24,063	1,992	5,097	12,737	1,043	3,988
CRAM 3.1 archive9	53,833	40,030	3,366	7,884	11,878	976	3,958
CRAM 4.0 normal	60,861	9,708	830	1,299	3,624	455	1,033
CRAM 4.0 small	55,514	19,723	1,635	2,625	12,472	1,018	2,041
CRAM 4.0 archive	53,679	22,238	1,843	5,078	11,651	949	4,010
CRAM 4.0 archive9	53,145	42,953	3,627	7,553	11,660	953	3,939
Deez (normal)	62,951	22,301	11,763	3,600	11,478	3,477	3,812
Deez q2	59,751	27,029	16,015	3,549	19,436	10,434	4,426
Deez q1	54,883	29,820	18,743	3,915	22,564	13,495	4,006
Genozip 12.0.34 fast	72,849	49,086	4,579	6,546	20,726	1,934	1,888
Genozip 12.0.34 (normal)	62,396	111,378	10,560	9,328	44,372	3,843	4,700
Genozip 13.0.5 fast	63,375	20,754	4,709	7,794	12,384	1,167	2,979
Genozip 13.0.5 (normal)	56,536	61,218	6,862	9,139	40,675	3,522	4,505
Genozip 13.0.5 best	54,934	80,442	9,180	44,365	43,478	3,774	36,955
MPEG-G (estimated)	58,444	N/A	*8,280	N/A	N/A	*3,990	N/A

Note: Deez -q1 lacks random access for quality values.
(*)MPEG-G timings are for a different system. See text for details.

5.3 PacBio CLR data

Source: ftp://ftp.1000genomes.ebi.ac.uk/vol1/ftp/technical/working/
20131209_na12878_pacbio/si/NA12878.pacbio.bwa-sw.20140202.bam

The document "Results_MPEG-G_Detailed_M56361.xlsx" states 13,766,776 records, The original BAM file has 25,968,256 records with 13,682,101 primary alignments and 12,286,155 secondary alignments. Given that secondary alignments share the same sequence and quality as their primary, it may be possible to deduplicate these. MPEG-G merges records from the same template together if they occur within the same Access Unit, which is stated to be blocks of 65,536 records. 68% of the secondary alignments are within 65,536 of their primary, so this cannot account for the disparity in the number of records. This document also states "BAM AUX: NM, MD, RG".

Dropping all secondary alignments and most auxiliary tags with `samtools view -F 0x100 -x AS -x PG -x SA -x XS` produces a BAM file closely matching the one quoted in M56361 (it is 0.7% smaller, but that may be due to Deflate implementation differences) and an even closer match to the CRAM 3.0 size (0.3% smaller). We attempted to clarify this, but received no answer. Therefore we are working on the assumption this transformation was applied and have done the same on our results for purposes of a fair comparison. Our results are valid between the tests we performed on versions of CRAM, Deez and Genozip, but we have listed MPEG-G sizes as "estimated" due to this lack of certainty.

Figure 5 shows the break down in data type sizes. As the sequences are long, read names are a tiny proportion of the total data. The PacBio qualities are dominant and hard to compress effectively. As explained above auxiliary tags were removed, but Genozip (default mode) still stored the MD:Z, NM:i and RG:Z tags. As in the HiSeq test, the data type sizes for MPEG-G come from the M56361 document. Although this was an earlier report, the total file size matches the one listed in their supplementary results so we believe them to be valid.

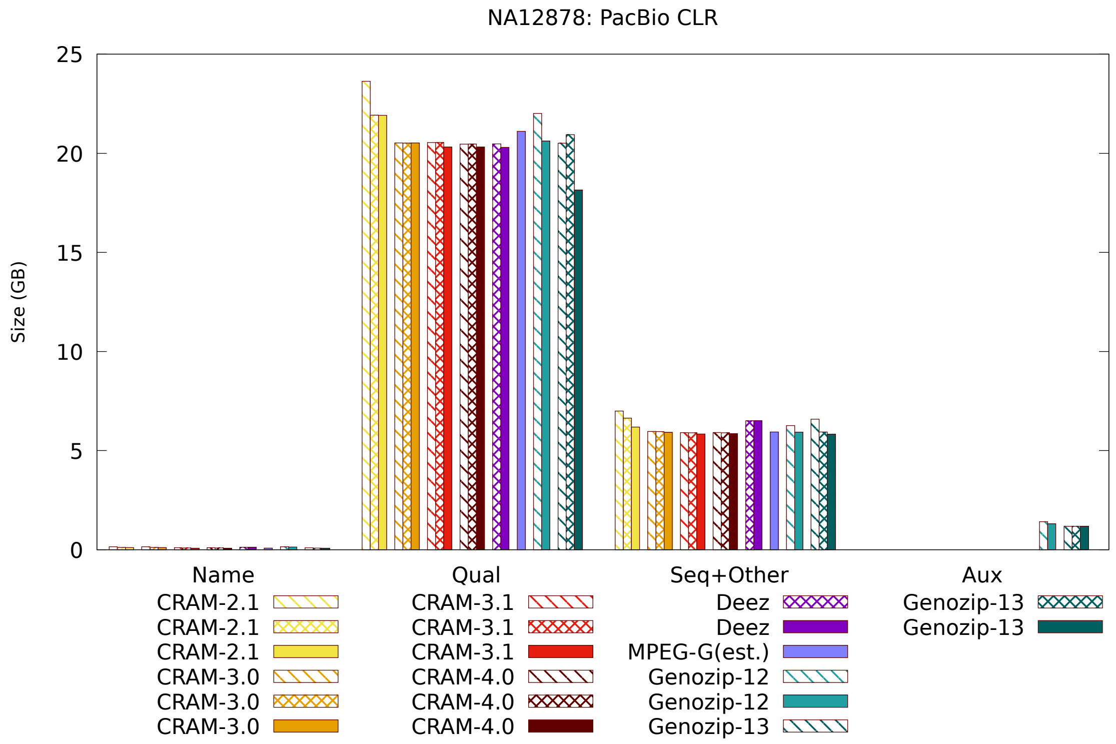

Figure 5: A break down of the relative size for each data type. Successive bars for the same format represent more advanced compression options. Genozip12 and Genozip13 refer to versions 12.0.34 and 13.0.5 respectively.

Of note here is this data set is dominated by the complexity of the quality scores. These are very random, without much variation between tools. The exception to this is the latest release of GenoZip which utilises the sequence as a context for compression of quality values. Such cross-correlation between data types is currently deliberately avoided in CRAM as it makes selective decode of individual data types impossible. However it is clear the gains may be significant and it is unlikely that there is a demand for obtaining quality values without also obtaining the sequences (unlike vice versa). Experiments (unpublished) demonstrate using a sequence context have a similarly considerable impact on PacBio CRAM quality, so this restriction could potentially be relaxed for CRAM 4.0, although the preferable route would be for the sequencing vendor to perform a significant quantisation to reduce the cost of quality encoding.

Unlike the HiSeq data, timings for the MPEG-G results come from the MPEG-G paper supplementary results and not the M56361 publication. Note these were performed using 8 threads on an Intel Core i7-7700 CPU running at 3.6 GHz. This is considerably different to our results using 12 cores on an Intel Xeon E5-2660 (nominally running at 2.2 GHz but with CPU frequency scaling sometimes temporarily overclocking this). However we note the HiSeq data set has speeds reported on both the i7-7700 system and the E5-2660 with encode times of 8,280 and 7,320 respectively and decode times of 3,990 and 3,745 respectively. This implies the MPEG-G timings below may be between 7 and 12% speed higher than if they were executed on the E5-2660 system. Although the MPEG-G timings are not directly comparable, we believe them to be broadly in the same ballpark and certainly within $\pm 50\%$.

We also repeated the PacBio CRAM 3.1 test on a different system with AVX512 support to test the fastest rANS implementations. This test was with 4 cores on an Intel Xeon Gold 6142 CPU (nominally at 2.6GHZ but with auto-scaling enabled). The reduced core count is to ensure a long enough test and to avoid I/O effects. Results averaging over 3 runs are presented in Table 11.

Time saved varies by data set and codec being used. This file has about 77% of file contents in the quality data series, so a SIMD order-1 rANS codec makes a significant difference (16 to 22%). However the slower archival compression profiles would not save nearly as much, nor will files containing a lot of long textual auxiliary tags (such as "XA:Z:"). A small sample of NovaSeq data showed a modest 4% speed gain. It was also observed that using many threads reduced the impact as other factors become more dominant. Furthermore, without CPU pinning the clock speed of CPUs running AVX512 was generally lower, reducing the potential gains.

Table 10: PacBio CLR detailed compression timings

Format	Size (MB)	Encode time(s) / Mem(MiB) CPU	Elapsed	Peak Mem	Decode time(s) / Mem(MiB) CPU	Elapsed	Peak Mem
BAM normal	45,158	2,661	483	27	524	72	14
BAM small	43,195	10,441	1,056	39	516	69	13
BAM archive	42,956	14,706	1,357	40	520	63	14
CRAM 2.1 normal	30,799	2,585	344	2,034	652	149	1,132
CRAM 2.1 small	28,697	8,030	688	6,048	3,959	332	2,180
CRAM 2.1 archive	28,566	11,907	1,003	16,782	3,337	288	5,194
CRAM 2.1 archive9	28,235	39,049	3,982	18,052	3,583	494	3,661
CRAM 3.0 normal	26,661	1,559	172	1,296	615	75	1,191
CRAM 3.0 small	26,617	1,821	176	4,904	697	81	2,557
CRAM 3.0 archive	26,589	3,604	344	12,743	792	104	5,206
CRAM 3.0 archive9	26,570	16,525	1,475	18,552	830	103	5,261
CRAM 3.1 normal	26,576	1,686	164	1,814	588	77	1,225
CRAM 3.1 small	26,554	1,995	187	5,803	592	79	2,895
CRAM 3.1 archive	26,513	6,681	603	14,483	1,036	120	6,205
CRAM 3.1 archive9	26,252	19,502	1,753	22,329	3,201	279	4,128
CRAM 4.0 normal	26,477	1,681	162	1,633	609	71	1,230
CRAM 4.0 small	26,458	2,035	188	5,279	631	73	2,496
CRAM 4.0 archive	26,422	6,683	612	9,847	1,060	107	6,154
CRAM 4.0 archive9	26,236	20,066	1,826	22,633	3,174	271	4,104
Deez (normal)	27,101	3,925	2,907	4,619	1,732	670	8,415
Deez q2	26,952	5,315	3,571	4,632	3,780	2,657	8,558
Deez q1	26,919	5,675	3,919	4,954	4,243	3,101	8,786
Genozip 12.0.34 fast	29,857	8,559	726	6,382	4,338	382	1,843
Genozip 12.0.34 (normal)	27,957	22,148	1,895	8,061	14,397	1,232	3,320
Genozip 13.0.5 fast	28,345	2,680	263	5,523	1,912	176	2,048
Genozip 13.0.5 (normal)	28,057	6,828	605	5,796	3,858	358	1,867
Genozip 13.0.5 best	25,255	12,429	1,113	34,680	9,315	833	20,432

Note: Deez -q1 lacks random access for quality values.
(*)MPEG-G timings are for a different system. See text for details.

Table 11: PacBio CLR AVX512 compression timings, using 4 threads

Format	Size (bytes)	Encode time(s) CPU	Elapsed	Decode time(s) CPU	Elapsed
CRAM 3.1 Scalar	26,574,431,640	1,044.8	274.8	380.7	96.5
CRAM 3.1 AVX512	26,578,918,156	858.4	236.1	319.7	82.0

6 CRAM compression: unaligned data

Sequencing data is typically generated in FASTQ. We would not consider this to be a suitable long term storage format as aligning or assembling the data both improves compression ratios and also substantially boosts the usefulness. However as an interim solution, data compression is still valuable.

Traditionally standard text compressors (such as gzip) have been used. There are a wide variety of dedicated FASTQ compressors, but we limit ourselves to evaluating the same multi-purpose encoders listed above with unaligned data.

The most naive solution is simply to generate an unaligned BAM, CRAM and related formats. This is a fast workable solution and better than straight FASTQ as it permits the presence of sample meta-data in the file header and has a rigorously defined way to attach additional per sequence meta-data, such as bar-coding, instead of exploiting the FASTQ comment section to create a format within a format.

Our data set is ERR174310_1.fastq.gz and ERR174310_2.fastq.gz from the MPEG-G data set 01-1.

The ERR174310_1.fastq.gz file is in NCBI SRA format, which unfortunately is a non-standard FASTQ variant where the original read identifier is in column 2. To correct this a fix the `samtools import` was made in PR#1485. The command to convert from fastq pairs to BAM is `samtools import -@8 -N -1 ERR174310_1.fastq.gz -2 ERR174310_2.fastq.gz -o ERR174310.u.bam`. The `samtools fastq` command can be used to reverse the process.

Table 12 shows encoding performance for various tools. The MPEG-G line here comes from their paper, so was on an Intel i7-7700. All others were benchmarked by us on an Intel Xeon Gold 6142 using 8 threads. This is a different system to the aligned data benchmarks above as the aligner needed more memory. Note `samtools import` currently has no multi-threaded decoding of FASTQ, so threading scalability is limited. However this is a tool chain implementation issue rather than a limitation of file formats so we expect this to improve.

Table 12: Compression of unaligned FASTQ

Format	Size (bytes)	CPU(s)	Elapsed(s)
FASTQ.bgzf	36,770,864,911	3,534	1,017
BAM	35,141,235,517	2,857	1,184
CRAM 3.0	25,310,280,282	1,379	701
CRAM 3.1	24,454,192,729	1,857	704
CRAM 3.1 small	23,230,961,730	3,883	725
MPEG-G no-ref	24,111,913,018	6,166	1,193
Genozip	22,930,340,845	11,182	1,419

MPEG-G timings taken from the Supplementary Data for their paper.
Genozip command line: `genozip -@8 ERR174310_[12].fastq.gz`.

It can be seen that storing FASTQ in unaligned BAM offers little benefit over gzip / bgzf, but using CRAM is a significant improvement due to the columnar nature of the format. This is further improved by the new codecs included in CRAM 3.1. Despite the poor multi-threading scalability of `samtools import`, it is competitive on speed. CRAM 3.1 was only slightly beaten by Genozip on size but ran in a fraction of the time. The default block size for Genozip is 16MB which corresponds to about 65,000 records. This is also comparable to the quoted block size for MPEG-G. CRAM 3.1 defaults to 10,000 short-read records for the normal profile, 25,000 for small profile and 100,000 for archive (not tested above).

A more advanced solution is to perform either a rapid sequence assembly, as originally proposed by Fritz *et al.* or use an existing known reference to do reference based alignment. Such methods do not need to be biologically rigorous as we are not attempting to get the best assembly or the best alignments, but simply to reduce the data volumes instead. There are many dedicated

FASTQ compressors using such techniques, but we limit our comparison to the same tools above that also support aligned data, with bgzf (parallel gzip) as a baseline.

Alignment / assembly considerably reduces the data size, but comes with an additional CPU cost. CRAM does not prescribe a specific method and SAMtools / HTSlib do not have this built in, however we can use very fast aligners for this purpose. (It should be noted that spending too much time on this strategy simply implies you should be converting to fully aligned BAM/CRAM anyway in order to leverage the utility of aligning or assembly rather than purely as a compression method.)

For this research we chose the SNAP aligner. SNAP also cannot cope with the non-standard SRA / ENA FASTQ variant and we also get better CPU performance if we naively align the data as interleaved single-ended data rather than pairs. As a pipeline we use `samtools import` to convert from two FASTQ files to uncompressed unaligned BAM, which is fed into SNAP aligner. The aligner outputs uncompressed aligned SAM, which is finally converted to CRAM. As we don't need fine grained random access on FASTQ, we also increased the number of sequences per CRAM slice to 100,000. We provide a crude `run_aligned.sh` script to demonstrate this process.

The SNAP aligner parameters have been chosen to optimise the speed, as we are not attempting to get good alignments, rather to improve compression ratios. Options were `snap-aligner single grch37.snap -t 8 --b -d 8 -D 0 -f -bam -map -o -sam -`. We used SNAP 1.0beta.24 as this gave better speed and size than the latest release.

Note this is very much a proof of principle as the resulting CRAM cannot be trivially converted back to FASTQ without some processing to split apart the two reads into their /1 and /2 components (given we aligned it without the "paired" option). However sufficient data exists to do this without data loss. The data has also been reordered slightly in large blocks due to the multi-threaded implementation in SNAP. This could be fixed without affecting the size and likely having a minimal impact on speed, but this work has not been done.

The ideal solution would be an aligner which can go directly from compressed FASTQ to compressed CRAM in a single process while retaining the exact file ordering.

Table 13: Compression of unaligned FASTQ

Format	Size (bytes)	CPU(s)	Elapsed(s)
FASTQ.bgzf	36,770,864,911	3,534	1,017
SNAP + BAM	41,296,355,569	11,701	1,481
SNAP + CRAM 3.0 normal	18,023,168,960	7,885	1,139
SNAP + CRAM 3.0 small 100k	17,783,348,994	9,845	1,481
SNAP + CRAM 3.1 normal	17,105,337,821	8,507	1,196
SNAP + CRAM 3.1 small 100k	15,290,593,086	10,331	1,370
SNAP + CRAM 3.1 small 500k	15,074,547,559	10,668	1,575
MPEG-G global assembly	16,053,119,238	N/A	5,820
Genozip with ref	15,061,744,296	17,868	3,842

MPEG-G timings taken from the Supplementary Data for their paper

Table 13 shows alignment and assembly based methods of FASTQ compression. The BAM file is larger than the unaligned BAM, due to storing chromosome name and position, mate-pairing information and auxiliary tags, without any benefits from reference based compression. CRAM shows a considerable improvement in compression ratios, with an expected elevated CPU cost. Note Genozip was ran using options `-@8 --pair --reference h38.snap` while SNAP was running on h37. Genozip gave the smallest file, but was considerably slower than CRAM and the same comments in the unaligned data test regarding block sizes apply.

Using SNAP + CRAM in this way is not the only combination and it is not a production-ready solution yet, however the results demonstrate that the concept of marrying a rapid approximate aligner to CRAM is a viable and performant solution without needing to change to a different encoding format for FASTQ data.

7 CRAM compression: Crumble lossy output

The SynDip synthetic diploid genome consisting of CHM1 and CHM13 samples has previously been evaluated for lossy-compression with the Crumble tool.

Unlike many tools, Crumble does not utilise its own file format. Rather it modifies the quality string via a simple experiment: if removing the quality values at a specific loci (after alignment) does not change the variant call and does not significantly alter the variant confidence, then these values can be set to a constant high value if they confirm the call or a constant low value if they disagree. The impact of this is a heavily skewed distribution of quality values with much greater predictability and compression ratios.

To evaluate CRAM 3.1 we compared CRAM 3.0 and CRAM 3.1 on the pre-crumble (original) BAM and the post-crumble BAM. These are shown in Tables 14 and 15. The data was Chromosome 1 of CHM1_CHM13_2.bam from ENA accession PRJEB13208.

Table 14: SynDip compression, pre-Crumble

Format	Size (bytes)	Quality	OQ:Z	Read-Names
BAM	13,017,816,377	N/A	N/A	N/A
CRAM 3.0	7,412,319,925	4,106,563,351	2,039,939,253	506,820,881
CRAM 3.0 small	7,251,254,040	4,099,877,724	2,038,910,887	417,349,414
CRAM 3.1	7,110,617,135	4,108,014,656	1,954,404,510	295,405,271
CRAM 3.1 small	6,740,573,443	3,834,028,984	1,944,155,206	288,739,462

The original data has been passed through GATK's quality score recalibration tool, with the original qualities copied to the OQ:Z BAM auxiliary tag and the updated qualities put into the QUAL field. It can be seen that this has dramatically increased the entropy of the quality values. Note we believe this approach is no longer part of the GATK best practice, but have no direct experience in this. However the data files are a real dataset.

The default CRAM 3.0 file is 43% smaller than the default BAM, and the default CRAM 3.1 is 4.1% smaller than the 3.0.

Table 15: SynDip compression, post-Crumble

Format	Size (bytes)	Quality	Read-Names
BAM	3,847,978,151	447,519,295	362,906,006
CRAM 3.0	944,003,579	234,945,688	49,496,197
CRAM 3.0 small	892,917,164	229,930,974	36,065,903
CRAM 3.0 archive9	849,921,368	227,566,075	32,465,280
CRAM 3.1	900,645,212	214,840,082	32,539,728
CRAM 3.1 small	862,661,875	212,046,488	27,979,249
CRAM 3.1 archive9	817,935,498	203,689,827	23,413,440
CRAM 4.0	891,445,170	215,634,168	29,256,258
CRAM 4.0 small	851,998,233	212,630,819	24,391,963
CRAM 4.0 archive9	801,384,897	193,674,218	19,812,538

Note for the default BAM mode we estimated the space taken by quality values and read-names by replacing these fields with "*" and measuring the impact on the BAM file size.

The Crumble output here is modifying quality scores as outlined above, but also discarding read names (via HTSlibs "lossy-names" option) where the read-pair falls entirely within a single CRAM slice, and removing the large OQ:Z auxiliary tag. The modified qualities have reduced in size by 19-fold.

It can be seen that all versions of CRAM now significantly outperform BAM by a large ratio ratio than pre-Crumble, saving between 75 and 79% of storage. CRAM 3.1 is between 3.4 and 4.6% smaller than the analogous CRAM 3.0 file, mirroring the relative gains we saw in the pre-Crumble data. Also included is the early prototype for CRAM 4.0, which slightly pushes this trend further.

For both pre- and post-crumble data CRAM 3.1's default compression level is on within 1% of or smaller than CRAM 3.0's small profile (using larger block sizes and bzip2), while also being faster (speed not shown above).

8 Random access

High data compression is useful, but it sometimes comes with a random access cost. As we make the blocks larger than the granularity of random access coarser, the compression ratio will improve.

The comparisons so far have been with mostly default options and different tools have very different default block sizes. For The NA12878 NovaSeq data set we have computed the average number of alignment record for block as reported by the tools. These can be seen in Table 16.

Table 16: Record numbers per file block

Format	No. Records
BAM	156
CRAM (normal)	10,000
CRAM small	25,000
CRAM archive9	100,000
Deez	216,015
Genozip	39,883
MPEG-G	125,763

BAM record count was computed by dividing the uncompressed file by $0xff00$ (the size of a BGZF data block), and then dividing the total number of records by that figure. Similarly Deez and Genozip numbers are computed from the total record count and number of blocks. MPEG-G is reported to use 65,536 record pairs per Access Unit, and we counted the number of records observed per 65,536 uniq read identifiers. This is not quite a 2:1 ratio as some templates span access units or are aligned far away.

To test the I/O performance with random access, we performed a single range query on Chr1 at position 10,000,000 of length 1Kbp, 10Kbp, 100Kbp, 1Mbp and 10Mbp (ie position 10M to 20M). The average depth across the largest region was 36x. We used the `io_trace` tool to evaluate the number of read and seek system calls, and the total count of bytes transferred. We repeated the test using Unix `time` to measure the elapsed time to query and report SAM records, without threading enabled. These were piped into `wc -l` to validate the result. These results are presented in Table 17.

There were some disparities in the number of records reported, with Deez and Genozip reporting records that start on or after Chr1:10000000 rather than records that overlap. There is still some small discrepancy between these tools, but this was not investigated.

I/O stats are not visible for Deez as it uses memory-mapped I/O which cannot be seen in a system call trace. All reference based tools exclude the I/O on the reference itself (which is also mmaped for the HTSlib CRAM implementation). CRAM and BAM I/O figures did not include the index, while Genozip and Deez have an internal index. The index file size for BAM was 9,282,200 bytes and for CRAM it was 1,379,148 bytes.

A more complete comparison was made with BAM, CRAM 3.0 and CRAM 3.1 accessing all exon and all gene regions across Chromosome 1. The BED file was produced from Ensembl release 104 GTF at http://ftp.ensembl.org/pub/current_gtf/homo_sapiens/.

This was converted to exon and gene bed file with awk, and tested using the SAMtools multi-region iterator:

```
zcat Homo_sapiens.GRCh38.104.gtf.gz | awk '$1 == 1 && $3 == "gene" \
    {printf("chr%d\t%d\t%d\n", $1, $4-1, $5)}' > genes.bed
zcat Homo_sapiens.GRCh38.104.gtf.gz | awk '$1 == 1 && $3 == "exon" \
    {printf("chr%d\t%d\t%d\n", $1, $4-1, $5)}' > exons.bed

samtools view -f 0xffff -M -L exons.bed NA12878.final.bam
```

The genes and exons files have 5475 and 136680 regions respectively, covering 78.6Mbp and

Table 17: I/O figures per random access unit

Format	Region	Time(s)	Records	MBytes	Reads	Seeks
BAM	1Kbp	0.066	284	0.29	10	3
BAM	10Kbp	0.069	2,597	0.43	14	3
BAM	100Kbp	0.115	26,222	1.71	53	3
BAM	1Mbp	0.573	266,894	14.39	440	3
BAM	10Mbp	6.290	2,693,407	143.23	4,373	3
CRAM 3.1	1Kbp	0.047	284	0.36	10	1
CRAM 3.1	10Kbp	0.047	2,597	0.36	10	1
CRAM 3.1	100Kbp	0.116	26,222	0.72	20	1
CRAM 3.1	1Mbp	0.823	266,894	4.95	145	1
CRAM 3.1	10Mbp	8.115	2,693,407	49.22	1,361	1
Genozip 12.0.34	1Kbp	13.059	250	60.67	382	272
Genozip 12.0.34	10Kbp	13.595	2,563	61.48	426	335
Genozip 12.0.34	100Kb	13.636	26,188	61.48	426	335
Genozip 12.0.34	1Mbp	17.276	2,66,860	66.42	708	712
Genozip 12.0.34	10Mbp	53.834	26,93,373	114.60	3,476	4,443
Deez -q1	1Kbp	6.650	250	N/A	N/A	N/A
Deez -q1	10Kbp	6.537	2,562	N/A	N/A	N/A
Deez -q1	100Kbp	6.493	26,188	N/A	N/A	N/A
Deez -q1	1Mbp	7.682	266,859	N/A	N/A	N/A
Deez -q1	10Mbp	27.587	2,693,372	N/A	N/A	N/A

10.2Mbp of chromosome 1. Total I/O figures were achieved with io_trace:

```
io_trace -r `pwd` -- samtools view -f 0xffff -M -L exons.bed \
    `pwd`/NA12878.final.31.cram
```

Table 18 shows the I/O statistics for the exons and genes regions.

Table 18: I/O statistics when querying many regions

Format	Regions	Time(s)	Records	Data(MB)	Reads	Seeks
BAM	Exons	30.4	4,894,064	1,489.7	45,503	5,587
CRAM 3.1	Exons	65.6	4,894,064	844.6	23,017	580
CRAM 3.1 small	Exons	89.0	4,894,064	775.3	16,713	123
BAM	Genes	19.0	37,263,364	2,234.2	68,244	1,646
CRAM 3.1	Genes	71.6	37,263,364	910.4	24,559	331
CRAM 3.1 small	Genes	92.7	37,263,364	792.0	16,991	88

The I/O load is significantly lower for the CRAM files, but elapsed time was larger. Note these test files were in disk cache, so this mainly reflects CPU time. It is unknown what the elapsed time would be after purging from cache as we were unable to perform this experiment, but queries the genes.bed file on a different BAM out of disk cache showed 85s uncached and an I/O read throughput of 45MB/s. This would likely indicate on uncached data BAM is still faster than CRAM with local storage. For remote network based I/O, for example via an htsget server, we would expect this to be reversed due to fewer access regions (seeks), higher latencies and probable lower transfer speeds..
